# Polyurethane-functionalized starch nanocrystals as anti-tuberculosis drug carrier

**DOI:** 10.1038/s41598-021-86767-1

**Published:** 2021-04-15

**Authors:** Shivang K. Desai, Dhananjoy Mondal, Smritilekha Bera

**Affiliations:** School of Chemical Sciences, Central University of Gujarat, Gandhinagar, 382030 India

**Keywords:** Drug discovery, Chemistry, Materials science, Nanoscience and technology

## Abstract

Studies related to loading ability and delivery of clinically used first-line anti-tuberculosis drugs (ATDs) such as isoniazid, rifampicin, pyrazinamide and streptomycin on the surface of starch-derived bulk and nanopolyurethanes (SBPUs and SNPUs) as drug delivery systems (DDS) have been focused to minimise or remove the drug-associated adverse effects. The efficiencies of nanopolyurethanes obtained from the differently substituted cyclic aliphatic and aromatic isocyanates have been studied for drug loading and release purposes. Different advanced instrumental techniques analysed the structural and morphological properties, thermal stability and crystallinity of the starch nanopolyurethans. Average particle sizes ranging from 27.35–42.38 nm to 126.89–218.60 nm for starch nanopolyurethans, SNPU**3i** and SNPU**4i**, respectively, were determined by high-resolution transmission electron microscopy. Similarly, the loading efficiency of ATDs to the surfaces of SNPUs and SBPUs was observed in the range of 60–97% while ATDs-loaded SNPUs showed a sustainable release profile for all ATDs except for streptomycin. However, most SBPUs provided burst-release for all the above-mentioned ATDs in pH-dependent studies. The anti-tuberculosis assay against the *Mycobacterium tuberculosis* H37Rv strain revealed that streptomycin-loaded SNPU**4i** and isoniazid-loaded SNPU**7i** are approximately 42 and 7 times more active than the native streptomycin and isoniazid, respectively.

## Introduction

All over the world, community-associated tuberculosis caused by *Mycobacterium tuberculosis* is one of the most infectious diseases^1–3^. The clinically used anti-tuberculosis drugs requires prolonged and intensive treatment time leading to the generation of either non-compliance with an increased possibility of relapse of tuberculosis (TB) or even more severe multidrug-resistant (MDR) and extensively drug-resistant (XDR) TB to a patient due to genetic and molecular structural changes^4–6^. The process of developing new and improved drugs for the treatment of drug-resistant tuberculosis is exorbitant and time-consuming. Rather, the inclusion of new drug delivery devices or improving the bioavailability, stability, target-oriented modification, drug distribution, metabolism, availability of drugs at the infected site, therapy time, drug resistance, and so on for the existing TB drugs would be more economical and beneficial. In this direction, polyurethanes-based versatile materials, which are one of the most ubiquitously applied polymers in different fields, may be explored as a drug delivery system (DDS)^7^ for anti-tuberculosis drug delivery.

Different polyurethanes with a wide range of physicochemical properties are synthesized by varying either the reactant partners or their stoichiometric ratios. Polyurethane-based drug delivery systems may be applied for sustained release of the clinically used TB drugs. Thus, for this purpose, urethane-functionalised starch may be explored as the said modification may improve the properties of the native starch such as pore size, binding sites and volume; generally, these properties are very much vital for the inclusion of water, drug molecules and foreign bodies. Nowadays, the semi-synthetic nanostructured polymeric materials as drug vehicles required for many targeted therapies have been emerging as a frontier research area in pharmaceutical and biopharmaceutical field. For a drug to act in the body, it is supposed to be protected from the degradation (enzymatic or chemical) before reaching the targeted site in physiological condition. In this context, polymeric nanoparticle-based vehicles might be appropriate to act as a safeguard of the drug molecules. Not only that, the nanotechnology-based system improved higher payload capacity and ability to incorporate both lipophilic and hydrophilic drug substances according to the administration routes, such as oral, topical, parenteral, pulmonary and so forth^8–10^. Generally, the natural improvement of the pharmacokinetics of nanoparticles to increase the clearance time from blood has been achieved by tuning their sizes, shapes and surface modification etc*.* resulting the facilitation in penetration of the drugs into the cellular level. These nanoparticles as a drug carrier can control the sustained release, which indirectly improves the drug bioavailability enabling a reduction in the dose as well as in dosing frequency in addition to an improvement in patient compliance^11^.

A controlled-release system constructed with biocompatible nanoparticles is the essential features for the innovative drug delivery approach for the existing ATDs to fight against extensive infection of TB disease. In this scenario, carbohydrate-based nanoparticles^12–14^ are advantageous being biodegradable and ornamented with a large number of hydroxy or other hydrophilic groups, such as carboxyl groups, which may find useful for post-translational surface modification. Carbohydrate-derived nanoparticles particularly, starch-based nanoparticles (SNCs) find enormous applications in the drug delivery research. The SNCs-based drug vehicles can be designed for the delivery of the covalently and/or physically attached drug molecules to the infected sites^15–19^. The urethane-functionalized starch in bulk and nano state acted as potential drug delivery devices in the field of sustained-release of drug molecules^20–23^. Not only that, these covalently cross-linked polyurethane-based starch nanoparticles are capable to show the high load-carrying capacity of the drug in the physiological medium with improved stability and biodegradability^24–28^. Usually, in industries, cross-linking methods have been commonly used and ubiquitously applied for modifications of starch. As the drug loading and releasing efficiency depend on adsorption phenomena of the delivery systems, thus it varies based on surface morphologies and backbone moieties through hydrophilic and hydrophobic interactions. Herein it is relevant to mention that starch nanopolyurethans are found as a better adsorbent for biodiesel purification than that of simple SNCs as per or earlier studies^29,30^.

Herein, we have performed the chemical modifications in the structural framework of starch and starch nanocrystal for the formation of cross-linking using urethane functionalization to improve their stability, sorptivity, resilience and resistance against transformation, acid/base hydrolysis, enzymatic hydrolysis, reduction, oxidation and many other chemical processes. The different polyurethanes were synthesized using differently substituted aromatic, and cyclic aliphatic isocyanates and diisocyanates to introduce both hydrophobic and hydrophilic functionalities in the backbone of starch nanocrystals. Finally, the several challenges associated with traditional anti-tuberculosis drugs were addressed by utilizing the cross-linked starch polyurethanes as a nano and bulk carrier for the loading of first-line anti-tuberculosis drugs (ATDs), rifampicin (RIF), isoniazid (INH), pyrazinamide (PZA) and streptomycin (SM). Besides, the polyurethane-functionalized starch nanocrystals were also established as sustainable materials for improved drug delivery and their release kinetics. Overall, syntheses, loading capacity (LC), loading efficiency (LE), release behavior of the polyurethane-functionalized starch nanoparticles loaded with first-line ATDs and their biological efficacies against *M. smegmatis* and *M. tuberculosis* H37Rv strain have been studied.

## Results and discussion

### Native starch-based bulk polyurethanes (SBPUs)

**Scheme 1:**
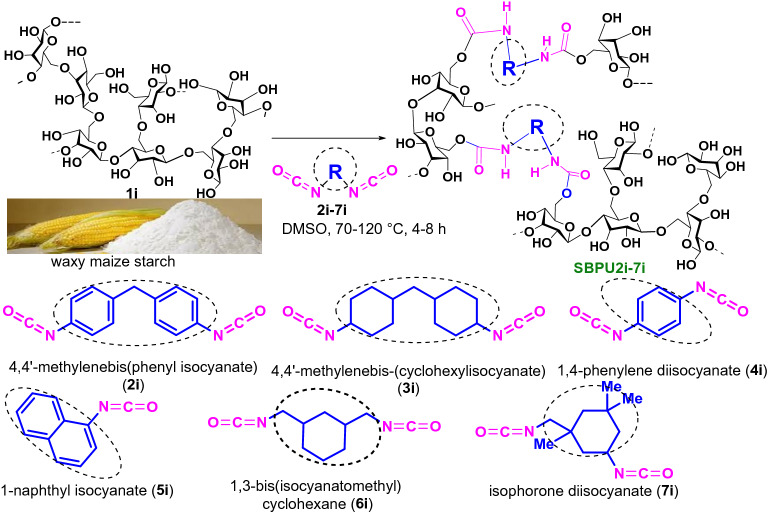
Synthesis of starch-based bulk polyurethanes (SBPU2i-7i).

The synthesis of starch-derived bulk polyurethanes (SBPUs) was completed by the reaction between native waxy maize starch (**1i**) and six different cyclic aliphatic/aromatic isocyanates such as 4,4′-methylene bis(phenyl isocyanate) (**2i**), 4,4′-methylene bis-(cyclohexyl isocyanate) (**3i**), 1,4-phenylene diisocyanate (**4i**), 1-naphthyl isocyanate (**5i**), 1,3-bis(isocyanatomethyl) cyclohexane (**6i**), and isophorone diisocyanate (**7i**) according to our previous report^29^. In brief, the waxy maize starch was first dried overnight in a high vacuum oven at 80 °C and then heated in DMSO at 55–60 °C for 24 h under constant stirring. After that, the isocyanate and an organometallic catalyst, stannous octoate was added and the mixture was stirred further at 70–120 °C, over 4–8 h (Scheme 1) depending on the nature of isocyanates. After completion of the reaction, cold methanol was added with constant stirring until the resulting polyurethane is precipitated. The solid residue was recovered through centrifugation at 8000 rpm and washed with acetone and water. Finally, the products (SBPU**2i-7i**) were dried in a vacuum oven at 80 °C for 24 h and stored in vacuum desiccator for further use. The yields of the reactions are reported in between 30 and 40%. Chemical structures of the SBPUs were corroborated by ^1^H NMR and FT-IR. ^1^H NMR signals corresponding to –NH group of the urethane linkages [–NH–(CO)–O–] and starch backbone of SBPU**5i**-7**i** were observed in the range of δ 7.1–8.5 and δ 3.0–5.8 ppm, respectively (Figs. S13 to S15). Whereas, for the case of SBPU**6i-7i** obtained from cyclic aliphatic isocyanates, the methylene protons of the cyclic backbone were observed in the range of ~ δ 2.63–0.73 ppm (Figs. S14 and S15). The amide bond [–(CO)–NH–] and urethane carbonyl corresponding to the urethane linkages are also confirmed by the FTIR spectroscopy; the sharp signals corresponding to amide bond and urethane carbonyl were detected at 1549 to 1559 cm^−1^ and 1655 to 1712 cm^−1^, respectively (Fig. S19). Chemical structures of the other polyurethane derivatives (SBPU**2i-4i)** have been reported in our previous work^29^.

**Scheme 2:**
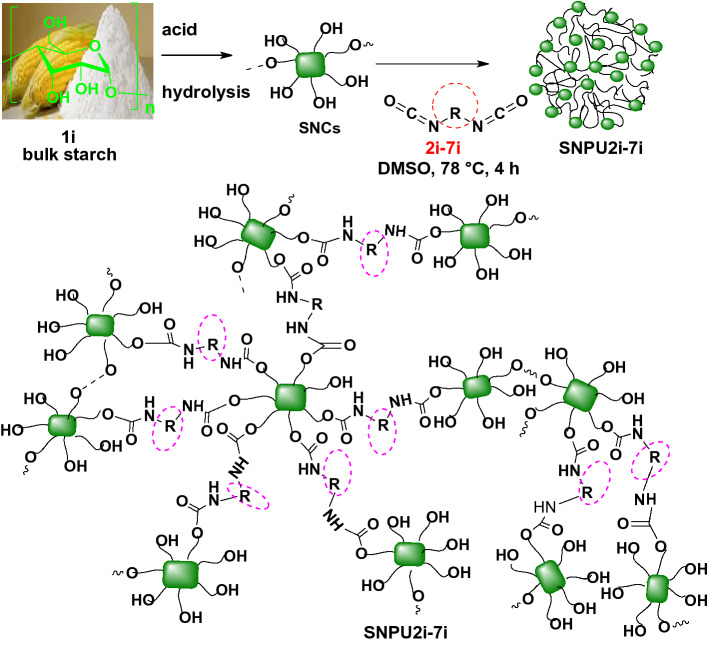
Synthesis of starch nanocrystal (SNCs) and starch-templated nanopolyurethanes (SNPUs).

Starch nanocrystal (SNCs) were first prepared according to our previous method^29^ from native waxy maize starch by acid hydrolysis with H_2_SO_4_ and purified by washing with distilled water for the synthesis of starch-derived nano polyurethanes (SNPUs). Furthermore, the resulting product was lyophilized for 36 h to render fine SNC powder in a fluffy white state. In order to stop their normal agglomeration, the SNCs were then suspended in chloroform and kept at 4° C. The average particle size and polydispersity index (PDI) of SNCs were found to be 312.44 nm and 0.425, respectively, in which due to its intrinsic agglomeration/aggregation characteristics the formation of the larger size of the SNC was realised. Using various mono and diisocyanates, the carbohydrate backbone of SNCs was cross-linked by urethane formation. In short, the SNCs were dispersed for 4 h at 55–60 °C in DMSO and then a similar reaction was carried out following the process previously used for bulk polyurethane synthesis (Scheme 2). For the formation of nanopolyurethanes, solvent displacements and precipitation methods have been used. The solids were recovered by centrifugation and washing with water/acetone mixture to produce **SNPU2i-7i,** which were dried in a vacuum oven and stored in vacuum desiccators for further use.

**Figure 1:**
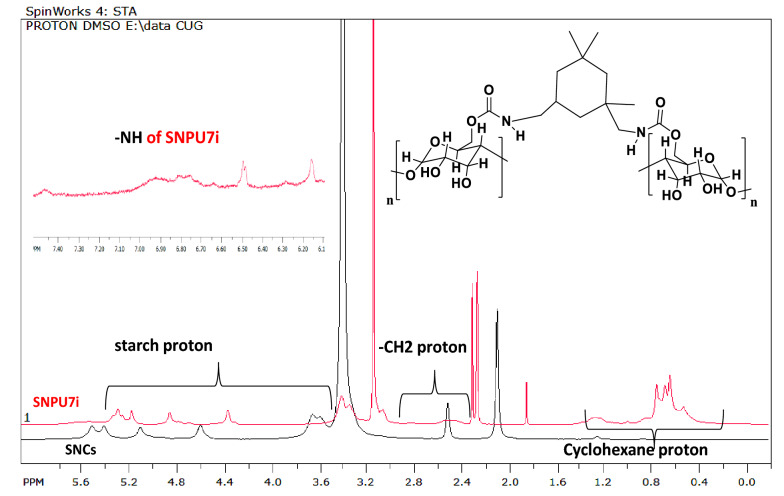
The ^1^H NMR of SNC and SNPU**7i** in DMSO-d6 at 500 MHz.

The structures of all cross-linked starch nanopolyurethanes (SNPU**2i** to SNPU**7i**) have been confirmed by NMR, FTIR, DLS, HR-TEM, P-XRD, TGA and other spectroscopic techniques. The peak between δ3.03–5.86 ppm corresponded to starch protons in the ^1^H NMR of SNCs and the relevant ^13^C signals are seen between δ78.4–71.2 ppm. Starch-derived nanopolyurethanes (SNPUs) contain urethanic bonds [–NH–(CO)–O–], confirmed by the NH peak in the range of δ 7.04–8.52 ppm in ^1^HNMR^29^ spectrum (Fig. S16 to S18); in Fig. 1, one of the examples (SNPU**2i**) is illustrated. In FTIR spectroscopy, the broad peak of hydroxy functionalities in starch at around 3200–3500 cm^−1^ was reduced upon isocyanate reaction, and peaks appeared at 1639.2 cm^−1^ and 1700 cm^−1^ corresponding to –C–NH and –C=O stretching vibrations of urethane linkages [-NH-(CO)-O-] along with other peaks of nano polyurethanes. A comparative study showed that the disappearance or the low intensity of the -NCO peak at ~ 2200 cm^−1^ suggested the use of isocyanates in the reaction (Fig. S20).

**Figure 2:**
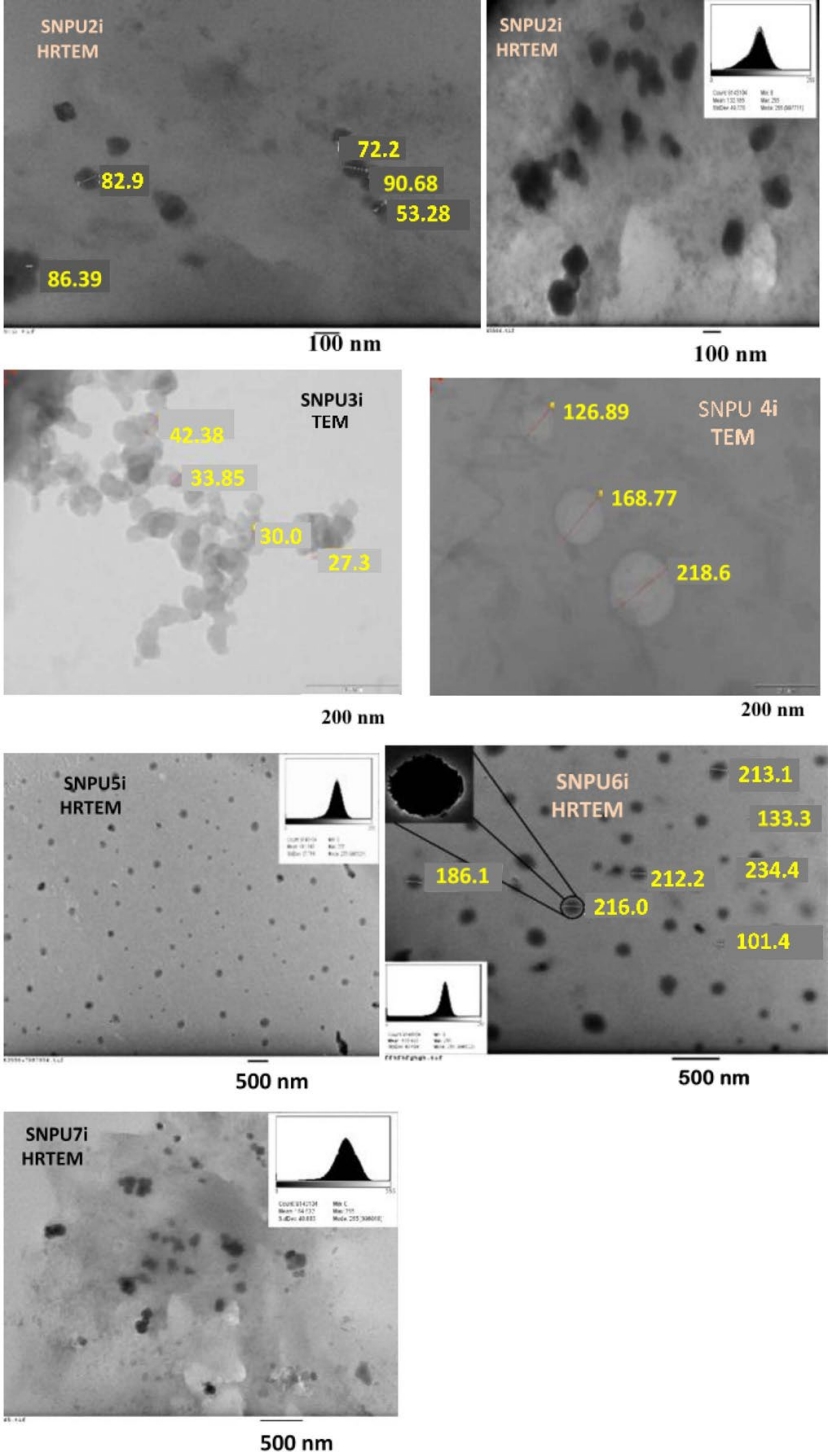
TEM and HRTEM images of starch nanopolyurethanes (**SNPU2i-7i).**

The average particle size of SNCs-based nanopolyurethanes (SNPU**2i-7i**) was measured by dynamic light scattering (DLS) and found in the range of 61–482 nm. The polydispersity index (PDI) of the polymer ranges from 0.292 to 1.313, suggesting a narrow distribution of particle sizes (Fig. S3 and Table S1). The size and morphology of the SNPU**3i-4i** polymers were characterised by TEM, which showed that the size of the SNPU**3i** and SNPU**4i** polymers was in the range of 27.35–42.38 nm and 126.89–218.60 nm, respectively. From the HRTEM image of SNPU**2i** (Fig. 2), it is evident that the particle is spherical in shape and the mean size is ~ 132.18 nm (standard deviation ~ 49 nm), while the mean size of the spherical-shaped nanopolyrethanes (SNPU**5i-7i**) is in the range of 160 to 170 nm (standard deviation ~ 42 nm).

**Figure 3:**
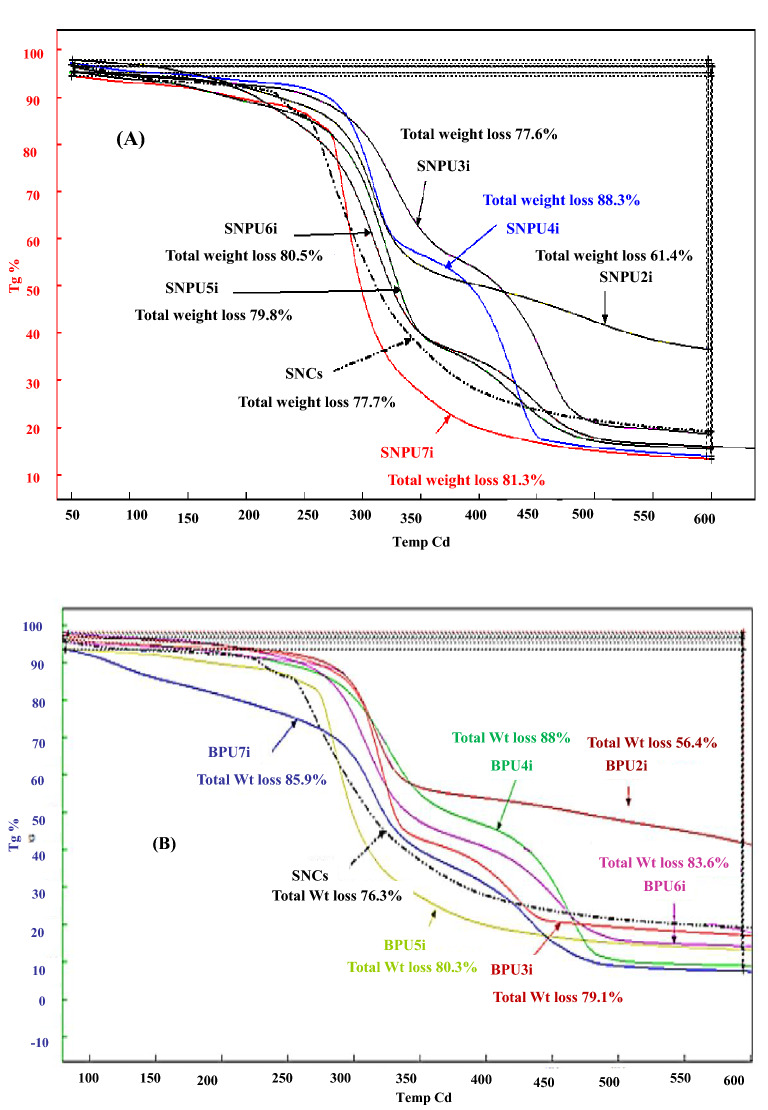
TGA thermograms of (**A**) nanopolyurethanes (SNPU**2i-7i**) with starch nanocrystal (SNC) and (**B**) bulk-polyurethanes (SBPU**2i-7i**) with native starch.

Thermal properties of the bulk and nanopolyurethanes (SBPU**2i-7i** and SNPU**2i-7i**)^31–34^ studied by TGA thermograms have shown that the thermal decomposition of starch nanocrystals varies from the starch-based bulk and nanopolyurethanes due to structural changes such as crystallinity and thus confirmed the grafting of the urethane linkages to native structure of SNCs (Fig. 3).

**Figure 4:**
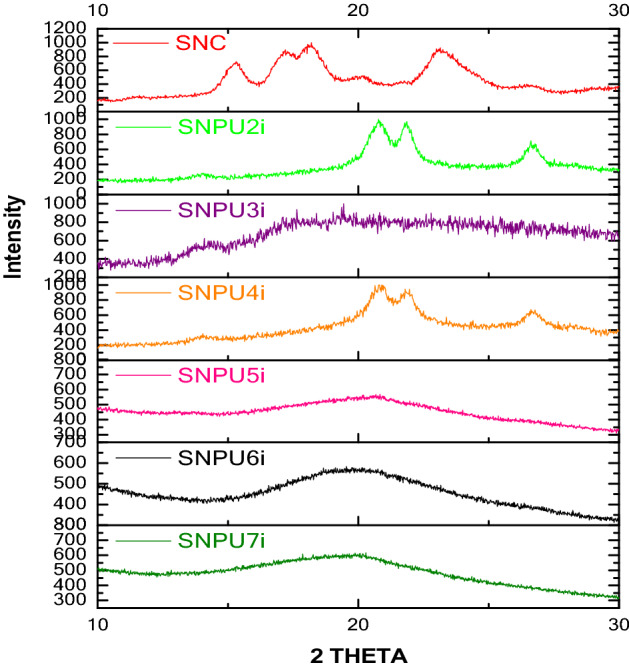
P-XRD spectra of nanopolyurethanes (**SNPU2i-7i**) with starch nanocrystals (SNCs).

The changes in crystallinity of polyurethanes as detected by TGA were also corroborated from the crystallinity pattern of SNCs-based polyurethanes investigated by P-XRD. The crystallinity pattern of SNC indicated four intense X-ray diffraction peaks at 15, 17, 18 and 23° 2θ. The cross-linking of SNC using urethane linkages reduces the crystallinity in polysaccharides by the changes in intensity and shifting of peaks. In the case of starch-based nanopolyurethanes, 2 theta values from 18 to 20° of pure SNCs was disappeared due to the formation of urethane linkages between starch and diisocyanates. From the literature precedents, it is found that the crystallinity is directly proportional to the cross-linking of diisocyanates with the polysaccharides of SNCs, and hence in case of modified SNCs-based PUs, less amount of crystalline domains are developed (Fig. 4). Among all SNPU**2i-7i**, the new crystalline domain was observed for SNPU**2i** and SNPU**4i** and for others; the changes in intensity and shifting of 2θ values indicated the non-crystalline or amorphous molecular arrangement. Since the urethane linkages are symmetrical in both of the cases, the presence of aromatic moiety might assist in the π-stacking interaction for the formation of crystalline nature.

### Loading of ATDs on starch-derived bulk and nanopolyurethanes (SBPU2i-7i and SNPU2i-7i)

**Figure 5:**
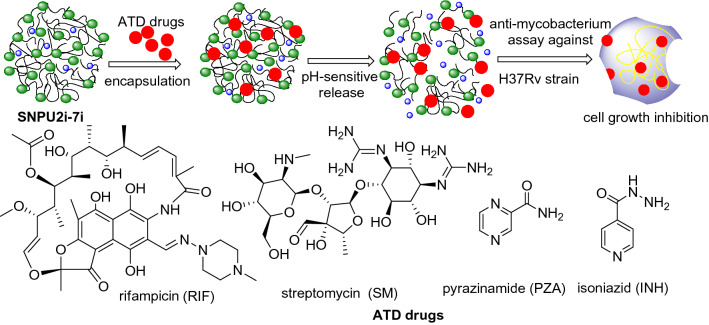
Starch-derived nanopolyurethanes (SNPU**2i-7i**) for drug delivery.

**Figure 6:**
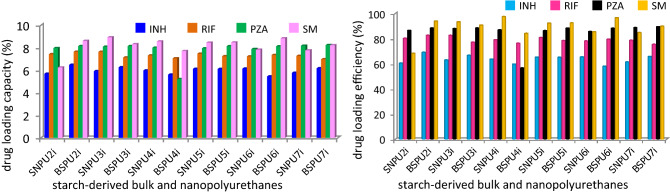
Drug loading capacity percentage (LC%) and efficiency percentage (LE%) of SBUPs**2i-7i** and SNPU**2i-7i** for streptomycin, isoniazid, pyrazinamide and rifampicin at 280, 270, 330 and 475 nm, respectively in THF using absorption spectroscopy.

The widespread use of nanoparticle-based carriers for the delivery of anti-tubercular drugs has become on priority due to the unique and advantageous physicochemical and pharmacokinetic profiles. Four first-line anti-tuberculosis drugs, such as rifampicin (RIF), isoniazid (INH), pyrazinamide (PZA), and streptomycin (SM) were considered for loading through physical adsorption on the surface of the starch-derived nano and bulk-polyurethanes (SNPU**2i-7i** and SBPU**2i-7i**); the drug-loaded polyurethanes were used for the study of their efficacy in drug delivery (Fig. 5). The loading capacities of SNPU**2i-7i** and SBPU**2i-7i** for ATDs were determined using a specific amount (10.0 mg) of isoniazid, pyrazinamide, and rifampicin in tetrahydrofuran (1.5 mL) and streptomycin in mixture of methanol and water (1:1). 100 mg of SNPU**2i-7i** and SBPU**2i-7i** was suspended individually in 13.5 mL THF in a vial containing 1.5 mL of ATDs solution in THF. The resulting mixture was stirred at a moderate speed in dark condition at room temperature for overnight. Then ATDs-loaded polymer was centrifuged and filtered, followed by two times washing with THF to remove free drugs. The drug loading efficiency (amount of drug encapsulated for the initial amount of drug taken) and drug loading capacity (amount of drug encapsulated concerning the amount of polymer) was investigated. The ATDs-loaded nanopolyurethanes were dried at room temperature for 24 h incubation period and stored in a desiccator; the drug loading capacity was measured by absorption spectroscopy. The loading percentage of each drug was calculated from the observed λ_max_ of streptomycin, isoniazid, pyrazinamide and rifampicin at 280, 270, 330 and 475 nm, respectively (Figs. S1, S2). These findings also assisted in determination or calculation of the concentration of the unloaded drugs. After 24 h of stirring, the UV–Vis absorbance of the supernatant containing unloaded drugs was measured from the linear plot of the respective ATDs drug. Calibration curves were plotted using 1 to 10% (0.0067 to 0.067 mg/mL) ATDs (isoniazid, pyrazinamide and rifampicin) added for the loading in the polymer composites while due to low absorbance, the 10 to 100% (0.067 to 0.67 mg/mL) streptomycin was added for loading in methanol/water (1:1, v/v). Unloaded and loaded drug concentrations were calculated using absorption spectroscopy. The loading capacities (%) of SNPU**2i-7i** and SBPU**2i-7i** are found almost similar for pyrazinamide, rifampicin and streptomycin in the range of ~ 7.0 to 9.0%. In contrast, the same for isoniazid is found to be in the range of ~ 5.5 to 6.7% and the loading efficiency of SNPU**4i** for streptomycin is found as highest. However, the loading efficiency of ATDs to SNPUs and SBPUs was found almost similar ranging from 60 to 97% (Fig. 6).

The loading of ATDs to SBPU**2i-7i** (Figs. S21–S24) and SNPU**2i-7i** (Figs. S25–S28) was also supported by the FTIR spectroscopic data. After loading of ATDs on starch nanopolyurethanes, the changes in the FTIR spectra were observed for nanopolyurethanes, such as broadening, shifting and intensity differences of peaks due to formation of several hydrogen bonds and other molecular interactions with functional groups present in ATDs (such as –OH and –NH_2_, C=O, aromatic C=C, CH etc*.*).

**Figure 7:**
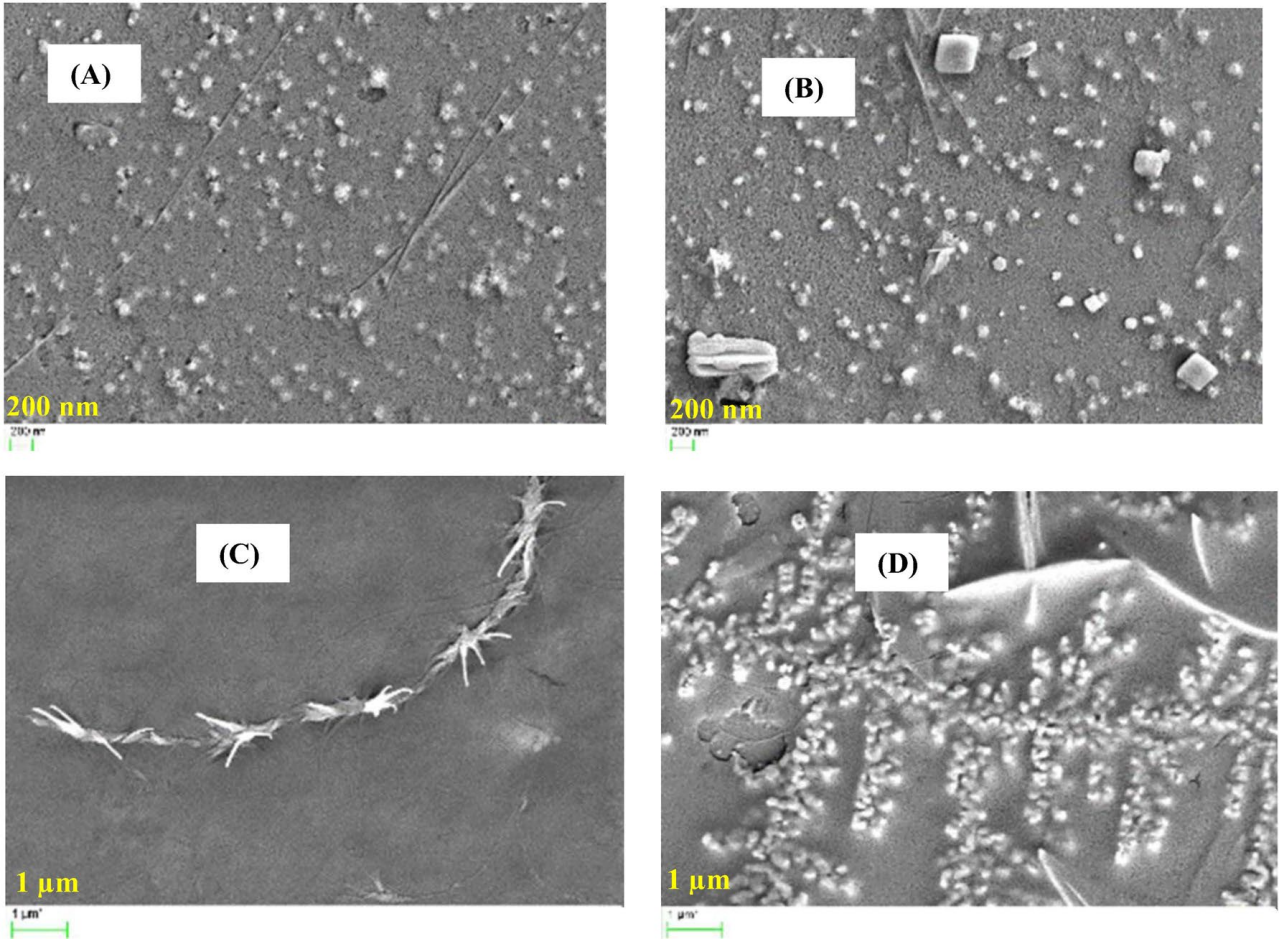
The FESEM images of (**A**) SNPU**2i** (**B**) PZA-loaded SNPU**2i** (**C**) SNPU**4i** (**D**) INH-loaded SNPU**4i.**

Besides that, the loading was also confirmed by FESEM images, which showed changes in polyurethane morphology before and after drug loading. FESEM images (Fig. 7) of nano polyurethanes loaded with isoniazid (INH) and pyrazinamide (PZA) showed that SNPU**2i** nanoparticles appeared as smooth and homogeneous spherical shapes prior to loading with pyrazinamide (Fig. 7A), but after loading they appeared to be irregular in shape (Fig. 7B). The changes in morphology before and after loading of isoniazid on SNPU**4i** are represented in Fig. 7C,D respectively. On loading of isoniazid, Fig. 7C showed the multi-branching chains and Fig. 7D displayed the spherical-shaped particle image embedded in the polymeric chain and branch.

### In vitro release studies of ATDs-loaded nano and bulk-polyurethanes

**Figure 8:**
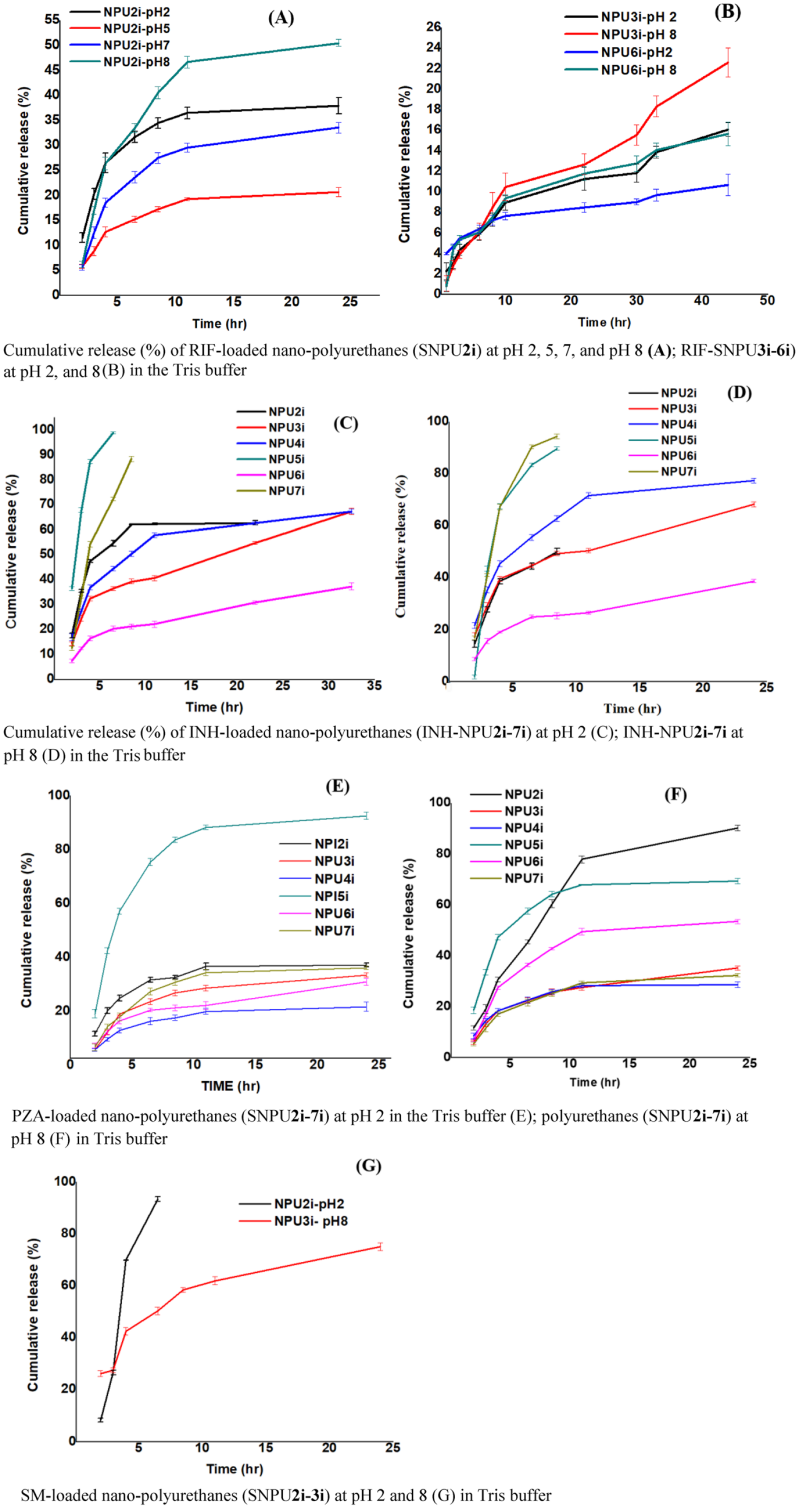
Cumulative release (%) of ATDs-loaded nanopolyurethanes (ATDs-SNPU**2i-7i**) at different in the Tris buffer *vs* time (**A–G**).

The in vitro release experiment of streptomycin, isoniazid, pyrazinamide and rifampicin using the ATDs-loaded SNPU**2i-7i** were performed by absorption spectroscopy at pH 2, 5, 7 and 8 in the Tris buffer. It was found that most of the drugs are released at pH 2 and pH 8 in the Tris buffer upon optimization. To assess the quantity of ATDs released from SNPU**2i-7i**, 25.0 mg of SNPU**2i-7i** loaded with ATDs were separately dispersed into various sample vials containing 10.0 mL of Tris buffer at pH 2 and pH 8 at 37 °C and stirred at 100 RPM. The 2.0 mL mixture solution of the individual release samples was applied to an equal volume of the corresponding Tris buffer at specific time intervals and the supernatants were analysed in triplicate by absorption spectroscopy. The percentage of cumulative drugs released at pH 2 and pH 8 in the Tris buffer was calculated at the respective λ_max_ of streptomycin, isoniazid, pyrazinamide and rifampicin (280, 270, 270 and 475 nm), respectively. For rifampicin-loaded SNPU**2i** at pH 2 and pH 8, the cumulative drug release percentage (CR%) was ~ 37 and 50%, respectively, within 24 h, while a maximum of 15 to 22% released was observed for SNPU**3i** and SNPU**6i** (Fig. 8A,B). Similarly, the CR% at pH 2 was found up to ~ 67% within 36 h in the case of isoniazid-loaded SNPU**2i-4i**, while SNPU**5i** and SNPU**7i** contributed up to 90% to burst release (Fig. 8C) at 8 h. CR % of isoniazid-loaded SNPU**3i**-4i provided a better result at pH 8 (CR % ~ 77%) compared to the result at pH 2 (Fig. 8C,D). The cumulative release percentage of pyrazinamide was found to be highest in case of pyrazinamide-loaded SNPU**5i** at pH 2 (CR% ~ 90%) while for pyrazinamide-loaded SNPU**5i** and SNPU**6i** at pH 8, CR% was ~ 70% and 54%, respectively, within 24 h. CR% of pyrazinamide for other pyrazinamide-loaded starch nanopolyurethanes (SNPU**3i,4i,6i,7i**) contributed in the range of 30–40% at pH 2 and pH 8 in Tris buffer (Fig. 8E,F).

**Figure 9:**
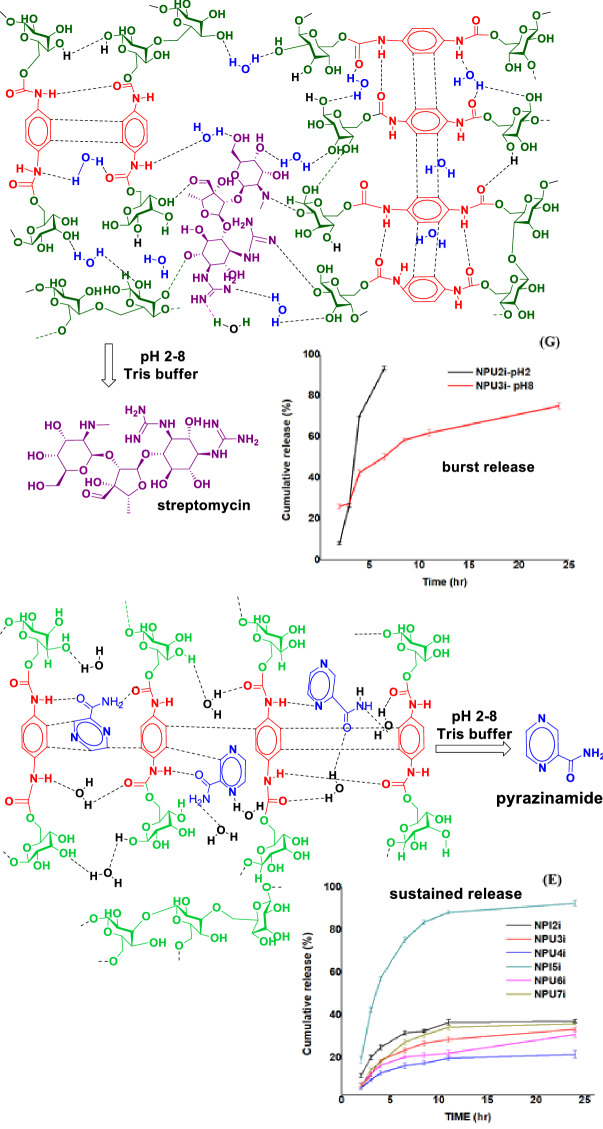
Mechanistic model of sustained and burst release.

For streptomycin-loaded nanopolyurethanes, no sustained release was observed; most of them developed burst release within a short time, suggesting that the drugs are mainly present in the nanosphere's outer side. Individually, streptomycin-loaded SNPU**3i** reported a cumulative streptomycin release of ~ 75% at pH 8 over 24 h (Fig. 8G). In Fig. 9, a mechanistic model was shown for burst release and sustained release. In general, the drugs are present outside the nanoparticle for burst release and the drugs find stability into the nanoparticle core through hydrogen bonding, van der Walls types of forces and/or electrostatic interactions for sustain release. Similarly, the release analysis of ATDs-loaded bulk-polyurethanes (SBPU**2i-7i**) at pH 2 and pH 8 in the Tris buffer showed that some of them have strong releasing properties other than pyrazinamide-loaded bulk-polyurethanes (Fig. S12A-H). The pyrazinamide-loaded SBPU**2i**, SBP**5i**, SBPU**7i** released the drug at pH 2 and pH 8 with moderate CR % in the range of ~ 30–45 percent. Isoniazid-loaded SBPU**3i** and SBPU**7i**, showed CR % up to ~ 69% for 30 h at pH 2, while other isoniazid-loaded SBPU**2i** and SBPU**4i-6i**, showed burst release at pH 2. CR % of isoniazid was observed up to 65% at pH 8 for SBPU**6i-7i**, while other bulk-polyurethanes SBPU**2i-5i** have shown burst release within a short period of time at pH 8. At pH 2 and 8 buffer, the cumulative release (%) of RIF-loaded bulk polyurethanes (SBPU**2i-7i**) contributed to a good rifampicin release profile. Rifampicin-loaded SBPU**4i** offered sustained release with CR% of rifampicin up to ~ 92% at pH 8 over 30 h duration, while sustained release of rifampicin was not found for other bulk polyurethanes in both media. Cumulative release (%) of streptomycin for streptomycin-loaded SBPU**2i** and SBPU**4i** showed better results with ~ 90% and 40% over 30 h’ time periods, respectively, while streptomycin-loaded other bulk polyurethanes provided burst release at pH 2 and 8 buffers.

The mathematical models for dissolution kinetic behavior of drug nanoparticles were studied to express the kinetics and release mechanism. Mathematical models were used to the solid dosage form for conducting drug release study and the results in Table 1 presented the comparative correlation coefficient (R^2^) of different kinetic models. The correlation coefficient (R^2^) is the prime factor in predicting the drug release mechanism through the best-fitted theoretical model. Table 1 shows the highest determination coefficient (R^2^) best fitted for zero-order, first-order, Higuchi, Hixson-Crowell and Korsmeyer-Peppas models for SNPU/SBPU**2i-7i** as the correlation coefficient value towards 1.0 indicates a perfect fit, and highly reliable for future forecasts using model, while a value of 0.0 would indicate that the calculation fails to accurately model the data at all. The zero-order model is best fitted for RIF-SNPU**2i**-pH8, RIF-SNPU**3i**-pH8, PZA-SNPU**2i**-pH8, RIF-SBPU**6i**-pH2, SM-SBPU**7i**-pH8 and this model shows the release mechanism through diffusion. The first-order modal is best fitted for RIF-SNPU**2i**-pH8, RIF-SNPU**3i**-pH8, INH-SNPU**4i**-pH2, INH-SNPU**4i**-pH8, PZA-SNPU**5i**-pH2, PZA-SNPU**2i**-pH8, PZA-SNPU**5i**-pH8, RIF-SBPU**6i**-pH2 and the release kinetic is explained by Fick’s first law of diffusion mechanism. While, Higuchi model is best fitted for INH-SNPU**2i**-pH2, INH-SNPU**3i**-pH2, INH-SNPU**4i**-pH2, INH-SNPU**3i**-pH8, INH-SNPU**4i**-pH8, PZA-SNPU**5i**-pH8, RIF-SBPU**4i**-pH8, and follows the diffusion medium-based mechanism according to Fick's first law. However, Hixson-Crowell model is the suitable for RIF-SNPU3i-pH8, INH-SNPU**2i**-pH2, INH-SNPU**4i**-pH2, INH-SNPU**3i**-pH8, INH-SNPU**4i**-pH8, PZA-SNPU**5i**-pH2, PZA-SNPU**2i**-pH8, RIF-SBPU**6i**-pH2, which explains the erosion release mechanism of drug release kinetics (Figs. S29–S43). In Table 1, various drug loaded carriers followed mixed mechanism of drug release such as diffusion, erosion, leaching, and Fick’s first law of diffusion mechanism etc^35–37^.

**Table 1:** Mathematical models for drug dissolution kinetics for NPU2i-7i.

	Zero-order	First-order	Higuchi	Hixson-Crowell	Korsmeyer-Peppas
RIF-NPU**2i**-pH8	0.9864	0.999	0.966	0.9657	0.9964
RIF-NPU**3i**-pH8	0.9921	0.9899	0.8934	0.9908	0.8835
SM-NPU**3i**-Ph8	0.885	0.9524	0.9789	0.9742	0.9337
INH-NPU**2i**-pH2	0.8328	0.8991	0.9379	0.9657	0.8793
INH-NPU**3i**-pH2	0.8029	0.8452	0.9449	0.8315	0.9544
INH-NPU**4i**-pH2	0.9155	0.9652	0.9768	0.9945	0.9543
INH-NPU**3i**-pH8	0.818	0.8789	0.9497	0.9617	0.8557
INH-NPU**4i**-pH8	0.9125	0.9839	0.9802	0.9951	0.9669
PZA-NPU**5i**-pH2	0.8944	0.9856	0.9408	0.9673	0.9856
PZA-NPU**2i**-pH8	0.9968	0.9563	0.8899	0.9799	0.9376
PZA-NPU**5i**-pH8	0.8807	0.9504	0.9576	0.9306	0.9856
RIF-BPU**6i**-pH2	0.9801	0.9781	0.9436	0.9831	0.9384
RIF-BPU**4i**-pH8	0.8697	0.9368	0.9497	0.9189	0.9611
SM-BPU**2i**-pH2	0.9347	0.9375	0.8563	0.936	0.8009
SM-BPU**7i**-pH8	0.9624	0.8924	0.7954	0.9207	0.8489

#### Anti-mycobacterial assay against *Mycobacterium smegmatis*

Prior to anti-tuberculosis assay, a rapid screening of the selected SBPUs was applied for detection of anti-mycobacterial activity against *Mycobacterium smegmatis* by disk diffusion assay. The streptomycin, isoniazid, and rifampicin-loaded SBPUs produced a positive result against *M. smegmatis* as illustrated in Table 2. The streptomycin-loaded SBPUs produced promising activity result against *M. smegmatis* where rifampicin was used as control.

**Table 2:** Anti-mycobacterial activity against *Mycobacterium smegmatis* by disk diffusion assay.

Experimental samples	Zone of inhibition (mm)
SM-SBPU**2i**	18 ± 2.3
RIF-SBPU**4i**	–
SM-SBPU**4i**	24 ± 1.9
INH-SBPU**3i**	–
SM-SBPU**3i**	22 ± 1.5
INH-SBPU**5i**	–
RIF-SBPU**5i**	–
INH-SBPU**6i**	–
RIF-SBPU**6i**	17 ± 2.7
SM-SBPU**6i**	–
INH-SBPU**7i**	–
RIF-SBPU**7i**	–
SM-SBPU**7i**	20 ± 2.9
Rifampicin (RIF)	15 ± 1.5

The diameter of the filter paper disks (5.0 mm) is included; (–) not active; values are mean ± standard deviation of three experiments in replicate.

### Anti-tuberculosis assay

Minimum inhibition concentration (MIC) was calculated to determine the anti-tuberculosis activity against *M. tuberculosis* strain H37Rv using the Lowenstein-Jensen (LJ) slope method, which is a non-automated in vitro bacterial susceptibility procedure. The protocol offers a quantitative outcome for the number of antimicrobial agents, required to inhibit the progression of a targeted microscopic organism. As a primary target, ATDs-loaded SNPU**2i-7i** were studied for the anti-tuberculosis efficacy against *M. tuberculosis* strain H37Rv. Surprisingly, nanopolyurethanes loaded with ATDs are 2 to 40 times more effective than standard ATDs. MIC of standard isoniazid and streptomycin are 0.20 and 0.90 μg/mL, respectively, against H37Rv strains. In the case of streptomycin-loaded SNPU**2i** (SM-SNPU**2i**), the MIC is 0.10 μg/mL, which is 9.0 times more powerful than the standard streptomycin having concentration 0.90 μg/mL with 99% inhibition. It is worthful to conclude that, streptomycin-loaded SNPU**4i** (SM-SNPU**4i**) is 42 times more effective than the native drug, which has proven to be one of the promising drug delivery systems (DDS) for streptomycin. Similarly, in contrast to the parent ATDs, isoniazid-loaded SNPUs (isoniazid-SNPU**3i, 5i-7i**) revealed better performance. Actually, isoniazid-loaded SNPU**6i**, **3i**, and **7i** worked 2, 6 and 7 times more effectively against H37Rv tuberculosis strains compared to that of native ATDs (Table 3). ATDs-loaded SNPUs have therefore acted as a powerful way to deliver systems for controlled and sustained drug release, which is an intriguing and promising feature that cannot be achieved with unloaded/free drugs.

**Table 3:** Anti-tuberculosis assay of ATDs-loaded SNPU2i-7i against H37Rv strains by LJ medium.

Experimental samples	MIC (μg/mL)	MIC (standard drug)
INH-SNPU**3i**	0.029	0.20 μg/mL (isoniazid)
INH-SNPU**5i**	1.90	
INH-SNPU**6i**	0.090	
INH-SNPU**7i**	0.028	
SM-SNPU**4i**	0.021	0.90 μg/mL (streptomycin)
SM-SNPU**2i**	0.10	
PZA-SNPU**5i**	0.039	75 μg/mL (pyrazinamide)
RIF-SBPU**4i**	17.6	0.09 μg/mL (rifampicin)
SM-SBPU**2i**	10.7	0.90 μg/mL (streptomycin)
SM-SBPU**3i**	5.18	
SM-SBPU**6i**	11.0	
SM-SBPU**7i**	10.2	

## Materials and methods

Commercially available, analytical-grade starting materials for the synthesis of carbohydrate-based nanopolyurethanes were purchased from Sigma-Aldrich, TCI, SRL, Rankem and used as received, unless otherwise noted. The FTIR was recorded on KBr pellets with a Perkin Elmer Model 1600 infrared spectrometer. The structure of the synthesised compound was confirmed by ^1^H NMR spectroscopy and reported in dimethyl sulfoxide-d6 (DMSO-d6) at room temperature on a Bruker Avance 500 MHz FTNMR spectrophotometer. ^1^H NMR data in the presence of internal standard (TMS, 0.0 ppm), chemical shift with multiplicity, coupling constants in Hz, and integration were recorded in ppm (δ). Measurement of the average particle size of the polymeric nanoparticle was performed by dynamic light scattering (DLS), model no Microtrac Zetatrac, U2771 at 25 °C. The morphological changes and size of the polyurethane nanoparticles were examined by the High Resolution Transmission Electron Microscope (HRTEM)/TEM, model no JEOL JEM 2100F at an accelerating voltage of 200 kV. Samples are prepared on a copper grid of carbon-coated wire mesh, with an optimal sample thickness of less than 100 nm. The morphology of the SNPUs and ATDs-loaded SNPUs was analyzed by field emission scanning electron microscopy (FESEM: ULTRA-55 system, India). Powder X-ray diffraction (P-XRD) measurements were carried out in a Rigaku-made Smart Lab 9 kW rotating anode X-ray diffractometer (Kyoto, Japan) using Cu-K_α_ radiation (λ = 1.5418 e´) at 40 kV and 100 mA at an ambient temperature of 2θ (2° min^−1^). Thermal decomposition of SNPUs was studied with by TG/DTA 73,000 (EXSTAR) (TGA). Drug loading and release of ATDs-loaded polymeric polyurethanes were studied by UV − vis absorption on a Spectro 2060 + UV − Visible spectrophotometer.

### General procedure for the preparation of nanopolyurethanes (SNPU2i-7i)

The dried SNCs (200 mg, 0.3 mmol, 1.0 equiv.) in 4.0 mL DMSO was heated at 55 °C for 4 h. Then, diisocyanate (**2i-7i**) (2.3 equiv.) was added in the mixture followed by stannous octoate (**8i**) (0.03 equiv.) was added dropwise as a catalyst, and the resulting solution was further stirred at 70–85 °C for 5–8 h. The reaction was monitored primarily by the presence of diisocyanates on a TLC plate. Then the cold methanol was added in excess amount until the resulting PU was precipitated. The solids were recovered by centrifugation and washed extensively with water followed by acetone. Finally, the product was dried in a vacuum oven for 24 h and stored in vacuum desiccators for further use.

### The characterization data for SBPU2i, SBPU3i, SBPU4i and SNPU2i, SNPU3i, SNPU4i^29^

**SBPU5i: **^**1**^**H NMR** (500 MHz, DMSO-d6): δ 5.46–2.06 (starch protons), δ 2.06–5.46 (starch protons), 7.50–8.24 (naphthalene ring Ar protons), 9.18 (NH) ppm; **FTIR:** ν cm^−1^ 3280 (O–H str., amide N–H), 2933 (C-H str.), 1719 (urethanic C=O str.), 1646 (CNH str.), 1504 (Ar. C=C str.), 1424 (C-N str.), 1152 (C–OH str.).

S**BPU6i: **^**1**^**H NMR** (500 MHz, DMSO-d6 δ 0.73–0.95 (exocyclic methylene protons), 1.13–2.63 (protons of the cyclic ring), 2.68–5.76 (starch protons) ppm; **FTIR:** ν cm^−1^ 3381 (O–H str., amide N–H), 2930, 2852 (C-H str.), 1715 (urethanic C=O str.), 1568 (CNH str.), 1568 (Ar. C=C str.), 1434 (C-N str.), 1156 (C–OH str.).

**SBPU7i: **^**1**^**H NMR** (500 MHz, DMSO-d6): δ 0.78–0.91 (exocyclic methylene protons), 1.23–2.07 (cyclic protons), 2.72–5.86 (starch protons), 7.03–7.15 (NH proton) ppm; **FTIR:** ν cm^−1^ 3288 (O–H str., amide N–H), 2953, 2915 (C-H str.), 1713 (urethanic C=O str.), 1559 (CNH str.), 1568 (Ar. C=C str.), 1446 (C-N str.), 1155 (C–OH str.).

**SNPU5i: **^**1**^**H NMR** (500 MHz, DMSO-d6 δ 2.15–5.60 (starch protons), 7.65–8.31(naphthalene ring Ar protons), 9.26 (NH). ppm; **FTIR:** ν cm^−1^ 3397 (O–H str., amide N–H), 2956, 2925, 2836 (C-H str.), 1742 (urethanic C=O str.), 1646 (CNH str.), 1506 (Ar. C=C str.), 1430 (C-N str.), 1157 (C–OH str.).

**SNPU6i: **^**1**^**H NMR** (500 MHz, DMSO-d6 δ 0.83–0.95 (exocyclic methylene protons), 1.35–2.22 (cyclic protons), 3.05–5.50 (starch protons), 6.85 (NH proton) ppm; **FTIR:** ν cm^−1^ 3420 (O–H str., amide N–H), 2956, 2925, 2846 (C-H str.), 1711 (urethanic C=O str.), 1639 (CNH str.), 1564 (Ar. C=C str.), 1455 (C-N str.), 1157 (C–OH str.).

**SNPU7i: **^**1**^**H NMR** (500 MHz, DMSO-d6 δ 0.75–0.91 (exocyclic methylene protons), 1.25–2.08 (cyclic protons), 2.73–5.83 (starch protons), 7.01–7.15 (NH proton) ppm; **FTIR:** ν cm^−1^ 3366 (O–H str., amide N–H), 2956, 2925, 2846 (C-H str.), 1702 (urethanic C=O str.), 1649 (CNH str.), 1560 (Ar. C=C str.), 1467 (C-N str.), 1154 (C–OH str.).

### Experimental procedure for preparation of ATDs-loaded starch nanopolyurethanes (SNPU2i-7i)

For the preparation of ATDs-loaded SNPU**2i-7i,** isoniazid, pyrazinamide and rifampicin (10.0 mg) was dissolved in 1.5 mL THF except streptomycin (10.0 mg), which was dissolved in 1.5 mL water/methanol mixture (1:1, v/v). Then it was added in 13.5 mL THF containing 100.0 mg of nano-PUs and stirred for 24 h at moderate speed. Then, loading efficiency was determined; a supernatant solution containing the amount of the free drug was taken out and UV–vis absorbance of all samples was measured to examine the unloaded drug concentration. Further, the loading capacity and loading efficiency were calculated.

### Solution preparation of ATDs-loaded starch nanopolyurethanes (SNPU2i-7i) for UV–vis absorbance spectroscopy

Loading capacity and loading efficiency of SNPUs for ATDs were calculated using UV–vis titration. A solution of 10.0 mg of isoniazid, pyrazinamide and rifampicin were dissolved separately in 15.0 mL THF and assumed as 100% (0.67 mg/mL) solutions and diluted it with THF to prepare 1 to 10% solution (0.0067 to 0.067 mg/mL) for INH and PZA, while for RIF, it was converted to10 to 100% (0.067 mg/mL to 0.67 mg/mL). In case of streptomycin (SM), 10.0 mg was dissolved in 15.0 mL water: methanol (1:1, v/v) mixture and considered as 100% solutions and diluted it with the mixture of methanol and water to prepare 10 to 100% solution (0.067 to 0.67 mg/mL). The supernatant of ATDs-loaded solutions was diluted to get absorbance data.

### Experimental procedure for release of ATDs-loaded starch-derived nano polyurethanes (SNPU2i-7i)

The release of ATDs-loaded nanopolyurethanes (SNPU**2i-7i**) was studied in Tris buffer at pH 2 and 8. An amount of 25.0 mg ATDs-loaded PUs was taken in a vial containing 10.0 mL Tris buffer solution and the mixture was stirred at room temperature at moderate speed. To determine the releasing efficiency, a specific amount of supernatant was withdrawn and analyzed using absorption spectroscopy, and an equal volume of fresh Tris buffer was added to the sample vial. The collected sample was analyzed by absorption spectroscopy for determination of release percentage of the drug.

### Solution preparation of ATDs-loaded starch nanopolyurethanes (SNPU2i-7i) for drug release study by UV–vis absorbance spectroscopy

Spectroscopic titration curve of the drug shows a plot of absorbance as a function of the different amount of ATDs. UV–vis absorbance spectra of ATDs were recorded using the same releasing medium with known concentration. UV–vis absorbance spectra were recorded with 1 to 10% (0.02 to 0.20 mg/mL) pyrazinamide, 1 to 10% (0.02 to 0.20 mg/mL) isoniazid, 1 to 50% (0.02 to 1.0 mg/mL) rifampicin, and 10 to 100% (0.2 to 2.0 mg/mL) streptomycin at pH 2 and 8 in Tris buffer. UV–vis spectra of supernatant were documented using the same medium as blank. All the data were measured at room temperature.

#### Anti-mycobacterial activity against *Mycobacterium smegmatis*

The fast-growing, acid-fast bacilli *Mycobacterium smegmatis* are used for the screening of potential samples for the assessment of anti-tuberculosis compounds^38,39^. The use of this rapidly growing acid-fast bacillus was advantageous over *M. tuberculosis* because anti-mycobacterial testing method of compounds against this organism is simple, economic and less tedious. Their rapid growth rate within 2–3 days and nonpathogenic nature made the handling safe and time-saving.

#### Method for in vitro anti-tuberculosis efficacy: minimum inhibition concentration by Lowenstein–Jensen (LJ) slope method

The ATDs-loaded SNPUs were used for anti-tuberculosis assay. Different types of controls like drugs, vehicles, agar, organisms, and known antibacterial drugs were applied for the study. Experimental samples and standard drugs were tested against the *M. tuberculosis* H37Rv cultures [Acid Fast Bacilli] and MDR strains (resistant to isoniazid and rifampicin). The LJ nutrient medium was used to grow and dilute the drug suspension, and the McFarland standard was followed for inoculum size on bacterial growth to test strain. Herein, the DMSO acted as diluents/vehicle to find the desired concentration of the synthesized anti-tuberculosis agents and standard drugs to test upon standard microbial strains.

### Statistical analysis

All acquired data were reproduced and reported as means ± standard deviation (SD). Origin was used to evaluate any significant differences in the experiments.

## Conclusions

We have successfully developed starch-derived bulk and nanopolyurethanes for utilizing as promising and biodegradable materials for delivery devices to improve the anti-tuberculosis therapeutic efficacy of commonly used first-line anti-tuberculosis drugs such as isoniazid, rifampicin, pyrazinamide and streptomycin. The structural and morphological features, thermal stability and crystalline quality of the experimental compounds were unraveled by various advanced instrumental techniques, such as NMR, FTIR, UV–Vis, DLS, TEM, HRTEM, FESEM, TGA and XRD. The SNPU**3i** and SNPU**4i** were synthesised with an average particle size ranging from 27.35–42.38 nm to 126.89–218.60 nm, respectively, from the six starch-derived nanopolyurethanes (SNPU**2i-7i**). The loading efficiency of anti-tuberculosis drugs on starch-derived nanopolyurethanes and native starch was moderate to good and was found in the 60–97%. For this analysis, the pH-dependent drug-releasing study showed that starch-derived nanopolyurethanes had a sustained release profile for all drug-loaded carriers except streptomycin; however, mostly burst-release was reported by starch-derived bulk polyurethanes. For pyrazinamide-loaded SNPU**5i**, the cumulative release of pyrazinamide (%) was up to ~ 93% at pH 2 and ~ 70% at pH 8; the cumulative drug release percentage for isoniazid at pH 8 was up to ~ 80%, while SNPU**2i** and SNPU**6i** exhibited ~ 90% and 54% cumulative release for isoniazid, respectively, over 24 h at pH 8. Apart from SNPU**3i**, which showed ~ 75% cumulative release of streptomycin at pH 8 over 24 h, most streptomycin-loaded nanopolyurethanes showed burst release within a short period of time. Primarily, the selected samples of SBPUs against *M. smegmatis*, with rifampicin as control, presented good results for streptomycin. Streptomycin-loaded nanopolyurethane (0.021 μg/mL) was found to be ~ 40 times more active than free streptomycin (0.90 μg/mL) itself in the anti-tuberculosis assay against the *Mycobacterium tuberculosis* H37Rv strain. In comparison, activity enhancement was demonstrated by isoniazid-loaded SNPUs and pyrazinamide-loaded SNPUs. INH-loaded SNPU**6i, 5i, 3i**, and **7i** served 2, 5, 6, and 7 times more effective against H37Rv tuberculosis strains and found as better anti-tuberculosis agents as compared to pure INH.

## Supplementary Information

Supplementary Information.

## References

[1] Daniel TM (2006). The history of tuberculosis. Respir. Med..

[2] Hunter R, Actor J (2019). The pathogenesis of post-primary tuberculosis A game-changer for vaccine development. Tuberculosis.

[3] Fogel N (2015). Tuberculosis: A disease without boundaries. Tuberculosis.

[4] Singh S, Mariappan T, Shankar R, Sarda N, Singh B (2001). A critical review of the probable reasons for the poor variable bioavailability of rifampicin from anti-tubercular fixed-dose combination (FDC) products, and the likely solutions to the problem. Int. J Pharm..

[5] Panchagnula R (2004). Fixed dose combinations for tuberculosis: Lessons learned from clinical, formulation and regulatory perspective. Methods Find Exp. Clin. Pharmacol..

[6] Addington WW (1979). Patient compliance: the most serious remaining problem in the control of tuberculosis in the United States. Chest.

[7] Desai, S., Bera, S. & Mondal. D. Multifaceted synthesis, properties and applications of polyurethanes and its composites. *Curr. Org. Chem.* **23**, 1–29, (2019). and reference therein.

[8] Garg T, Rath G, Murthy R (2015). Current nanotechnological approaches for an effective delivery of bioactive drug molecules to overcome drug resistance tuberculosis. Curr. Pharm. Des..

[9] Garg T, Bhandari S, Rath G, Goyal AK (2015). Current strategies for targeted delivery of bio-active drug molecules in the treatment of brain tumor. J. Drug Target..

[10] Malik R, Garg T, Goyal AK, Rath G (2016). Diacerein-Loaded novel gastroretentive nanofiber system using PLLA: Development and in vitro characterization. Artif. Cells Nanomed. Biotechnol..

[11] Navalakhe R, Nandedkar T (2007). Application of nanotechnology in biomedicine. Indian J. Exp. Biol..

[12] Bera S, Mondal D (2019). Insights of synthetic analogues of anti-leprosy. Bioorg. Med. Chem..

[13] Bera S, Mondal D (2019). A role for ultrasound in agents the fabrication of carbohydrate-supported nanomaterials. J. Ultrasound.

[14] Bera, S., Mondal, D. *Stimuli-Sensitive Nanomaterials for Antimicrobial Drug Delivery* (Ed. Prof. A. M. Grumezescu) 271–302 (Elsevier Inc., 2018)

[15] Aboutaleb E (2012). Improved antimycobacterial activity of rifampin using solid lipid nanoparticles. Int. Nano Lett..

[16] Leitzke S (1998). Rationale for and efficacy of prolonged-interval treatment using liposome-encapsulated amikacin in experimental Mycobacterium avium infection. Agents Chemother..

[17] Pandey R, Ahmed Z, Sharma S, Khuller G (2003). Nanoparticle encapsulated antitubercular drugs as a potential oral drug delivery system against murine tuberculosis. Tuberculosis.

[18] Dube A (2013). Multimodal nanoparticles that provide immunomodulation and intracellular drug delivery for infectious diseases. Nanomedicine.

[19] Burkeev MZ, Tazhbaev EM, Zhaparova LZ, Zhappar NK, Zhumagalieva TS (2016). Synthesis and characterization of poly (DL-lactic acid) nanoparticles loaded with the antituberculosis drug isoniazid. Pharm. Chem. J..

[20] Xu R, Manias E, Snyder AJ, Runt L (2002). Low permeability biomedical polyurethane nanocomposites. J. Biomater. Res..

[21] Xu R, Manias E, Snyder AJ, Runt L (2001). New biomedical poly (urethane urea)−layered silicate nanocomposites. Macromolecules.

[22] Anderson JM (1998). Recent advances in biomedical polyurethane biostability and biodegradation. Polym. Int..

[23] Gorna, K. & Gogolewski, S. Biodegradable polyurethanes for implants. II. In vitro degradation and calcification of materials from poly (ε‐caprolactone)–poly (ethylene oxide) diols and various chain extenders. *J. Biomed. Mater. Res.* **60**, 592–606 (2002). 10.1002/jbm.10100 11948518

[24] Cao, Y., Baiyisaiti, A., Wong, C.-W., Hsu, S.-H. & Qi, R. Polyurethane nanoparticle-loaded fenofibrate exerts inhibitory effects on nonalcoholic fatty liver disease in mice. *Mol. Pharm.* **15**, 4550−4557 (2018). 10.1021/acs.molpharmaceut.8b00548 30188729

[25] Muttil P (2007). Inhalable microparticles containing large payload of anti-tuberculosis drugs. Eur. J. Pharm. Sci..

[26] Bhosle, G. S., Nawale, L., Yeware, A. M., Sarkar, D. & Fernandes, M. Antibacterial and anti-TB tat-peptidomimetics with improved efficacy and half-life. *Eur. J. Med. Chem.* **152**, 358–369 (2018). 10.1016/j.ejmech.2018.04.039 29738954

[27] Solanki A, Das M, Thakore S (2018). A review on carbohydrate embedded polyurethanes: An emerging area in the scope of biomedical applications. Carbohydr. Polym..

[28] Ou CW, Su CH, Jeng US, Hsu SH (2014). Characterization of biodegradable polyurethane nanoparticles and thermally induced self assembly in water dispersion. ACS Appl. Mater. Interfaces.

[29] Desai, S. Bera, S., Mondal, D., Singh. M. Polyurethane-functionalized starch nanoparticles for the purification of biodiesel. *J. Appl. Polym. Sci. 134*, 44463 (2017).

[30] Desai S, Bera S, Mondal D, Singh M (2017). Better biodiesel: A nano-cleansing solution. Curr. Sci..

[31] Da Róz A, Curvelo A, Gandini A (2009). Preparation and characterization of cross-linked starch polyurethanes. Carbohydr. Polym..

[32] Liang R-C (2015). Gemini quaternary ammonium-incorporated biodegradable multiblock polyurethane micelles for brain drug delivery. RSC Adv..

[33] Freichels H (2013). mannose functionalized hydroxyethyl starch nanocapsules: En route to drug delivery systems with targeting properties. J. Mater. Chem. B.

[34] Kubota T (2013). Synthesis of new carbohydrate-based polyurethanes and their application in the purification of methyl esters (biodiesel). J. Polym. Res..

[35] Main mechanisms to control the drug release, Editor(s): Bruschi, M. L. *Strategies to Modify the Drug Release from Pharmaceutical Systems* 37–62 (Woodhead Publishing, 2015). ISBN 9780081000922.

[36] Mathematical models of drug release, Editor(s): Bruschi, M. L., *Strategies to Modify the Drug Release from Pharmaceutical Systems* 63–86 (Woodhead Publishing, 2015). ISBN 9780081000922.

[37] Fu Y, Kao WJ (2010). Drug release kinetics and transport mechanisms of nondegradable and degradable polymeric delivery systems. Expert Opin. Drug Deliv..

[38] Jimenez-Arellanes A, Meckes M, Ramirez R, Torres J, Luna-Herrera J (2003). Activity against multidrugresistant *Mycobacterium tuberculosis* in Mexican plants used to treat respiratory diseases. Phytother. Res..

[39] Gupta VK (2007). Anti-mycobacterial activity of lichens. Pharm. Biol..

