# Supplementary material for: Polyurethane-functionalized starch nanocrystals as anti-tuberculosis drug carrier
Source: Sci Rep. 2021 Apr 15;11:8331. doi: 10.1038/s41598-021-86767-1 (PMC8050055; doi:10.1038/s41598-021-86767-1)
Supplement: Supplementary file 1 — Supplementary Information. [file 41598_2021_86767_MOESM1_ESM.pdf]

<u>**Supporting Information**</u>

Polyurethane-Functionalized Starch Nanocrystals as Anti-Tuberculosis Drug Carrier

Shivang K. Desai, Dhananjoy Mondal* and Smritilekha Bera*

[a]School of Chemical Sciences, Central University of Gujarat, Gandhinagar-382030, India
Email: lekha026@yahoo.com /dhananjoym@yahoo.com

**Contents:**	Page No.
**Figures S1-S2:** Absorbance spectra of ATDs in different solvents and linear plot of absorbance *vs* concentration (%) of ATDs at different wavelengths	S2
**Figure S3:** DLS spectra of (A) SNPU**2i** (B) SNPU**3i** (C) SNPU**4i** (D) SNPU**5i** (E) SNPU**6i** (F) SNPU**7i**	S3
**Table S1:** The average particle size of nanopolyurethanes (SNPU**2i-7i**) by DLS study	S3
**Figures S4-S11:** Calibration curve for absorbance of i*n vitro* release of ATDs at pH 2 and pH 8 in the Tris buffer	S4-S6
**Figure S12:** Plotting of cumulative release percentage (CR%) of ATDs-loaded bulk-polyurethanes (SBPU**2i-7i**) *vs* time (h) in the Tris buffer at different pH	S7
**Method for Minimal Inhibition Concentration:** Lowenstein–Jensen (LJ) slope method for in vitro anti-tuberculosis efficacy	S8
**Figures S13-S15:** ^{1}H NMR spectra of SBPU**5i-7i** in DMSO-d6 at 500MHz	S9-S10
**Figures S16-S18:** ^{1}H NMR spectra of SNPU**5i-7i** in DMSO-d6 at 500MHz	S11-S12
**Figure S19:** FTIR spectra of SBPU**5i-7i**	S13
**Figure S20:** FTIR spectra of SNC compared with the FTIR spectra of respective isocyanate (**5i-7i**) and SNPU**5i-7i**	S14
**Figures S21-S24:** FTIR spectra of ATDs-loaded bulk starch polyurethanes	S15-S18
**Figures S25-S28:** FTIR spectra of ATDs-loaded starch nanopolyurethanes	S19-S22
**Figure S29-S43:** The graphs of Zero order, First order, Higuchi, Hixson-Crowell and Kors-peppas models are plotted for ATDs release kinetics from NPU/BPU2i-7i at pH2 and pH8	S22-S29
References for mathematical model graph plotting of Figure No.S29-S43	S29
**Table S2:** The loading efficiency and cumulative release percentage (CR%) of nanopolyurethanes (SNPU**2i-7i**) baulkpolyurethanes (SBPU**2i-7i**)	S30-31

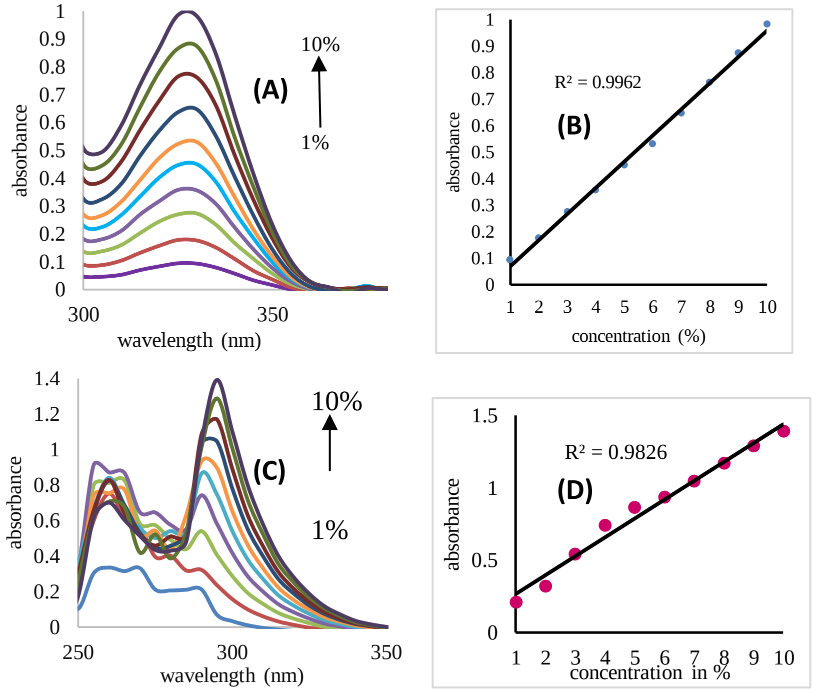

Figure S1. Absorbance spectra of ATDs in different solvents and linear plot of absorbance *vs* concentration (%) of ATDs at different wavelengths: (A) Absorbance spectra of pyrazinamide in THF; (B) linear plot of absorbance *vs* concentration (%) of pyrazinamide at 330 nm; (C) absorbance spectra of isoniazid in THF; (D) linear plot of absorbance *vs* concentration (%) of isoniazid at 290 nm.

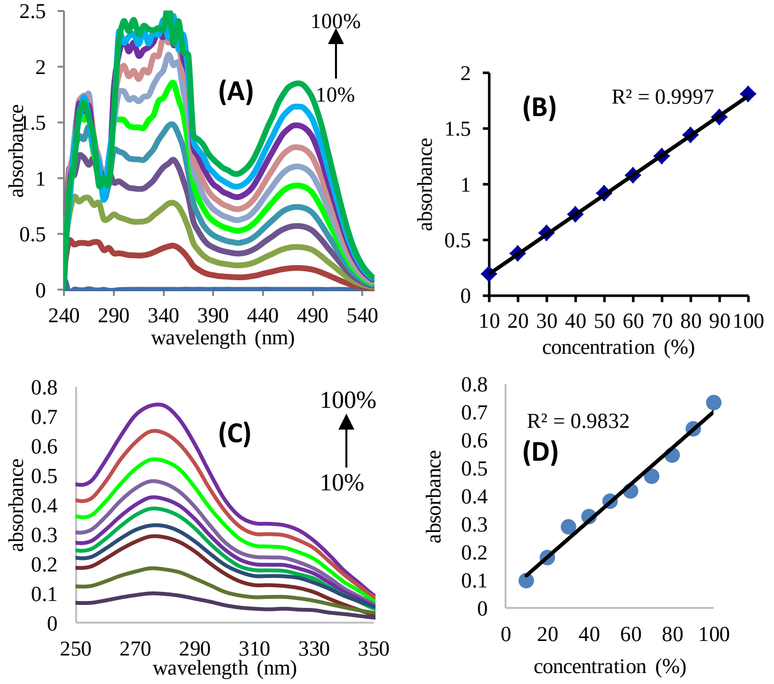

Figure S2. Absorbance spectra of ATDs in different solvents and linear plot of absorbance *vs* concentration (%) of ATDs at different wavelengths: (A) Absorbance spectra of rifampicin in THF; (B) linear plot of absorbance *vs* concentration (%) of

rifampicin at 475 nm; (C) streptomycin in water: methanol (1:1, v/v); (D) linear plot of absorbance vs concentration (%) of streptomycin at 280 nm.

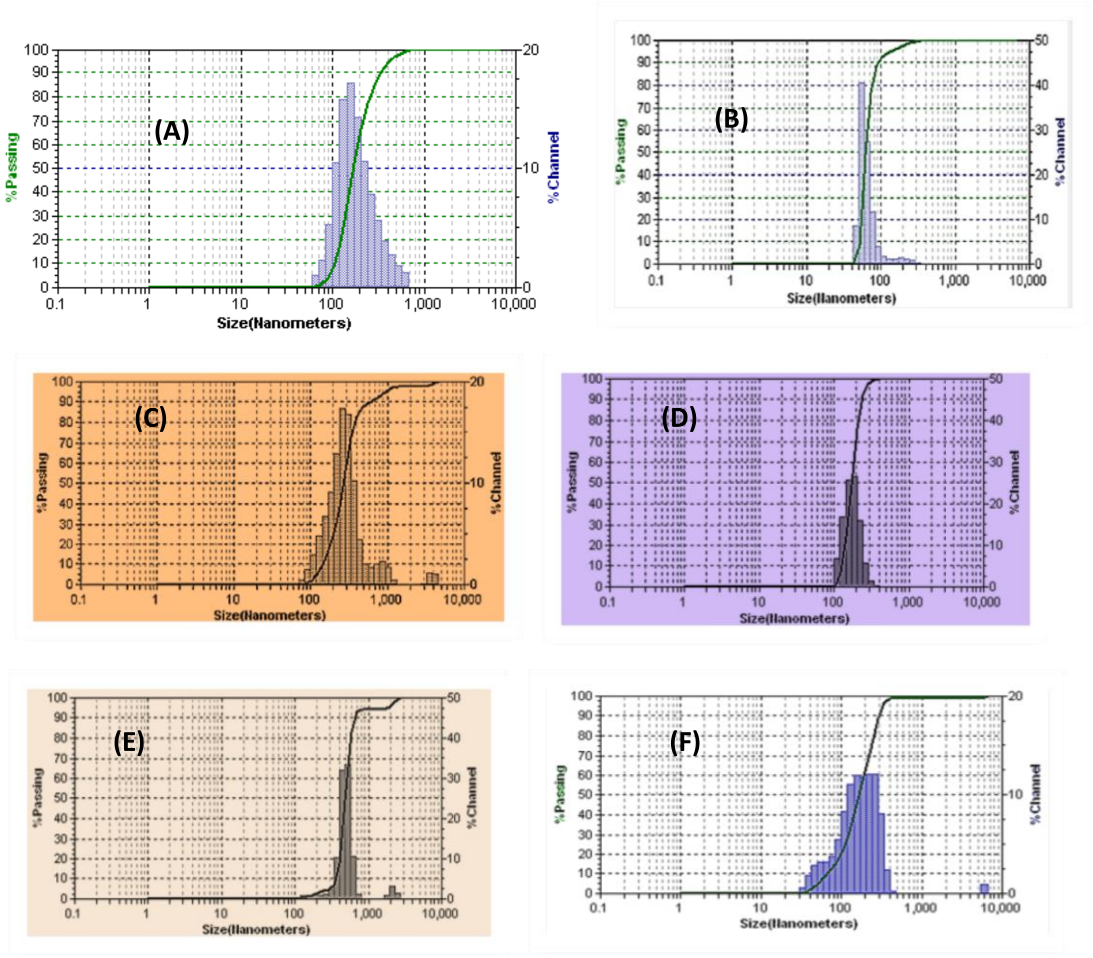

Figure S3. DLS spectra of (A) SNPU**2i** (B) SNPU**3i** (C) SNPU**4i** (D) SNPU**5i** (E) SNPU**6i** (F) SNPU**7i**

Table S1. The average particle size of nanopolyurethanes (S**NPU2i-7i**) by DLS study

**Nanopolyurethanes**	**PDI**	**Average particle size (nm)**
**(A)** SNPU**2i**	1.313	259.9 ± 251.2
**(B)** SNPU**3i**	0.957	61.0 ± 25.64
**(C)** SNPU**4i**	0.543	261.1 ± 198.4
**(D)** SNPU**5i**	0.374	172.5 ± 84.7
**(E)** SNPU**6i**	0.971	165.1 ± 181.3
**(F)** SNPU**7i**	0.292	482.0 ± 167.8

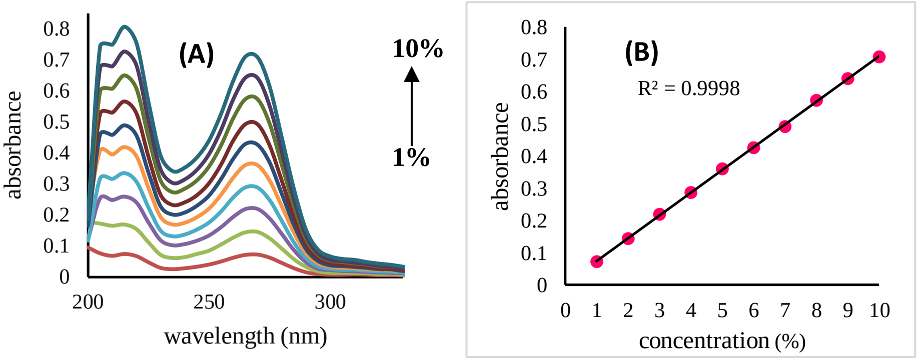

Figure S4. Calibration curve for absorbance of i*n vitro* release of ATDs at pH 2 in the Tris buffer: (A) Absorbance spectra of isoniazid at pH 2; (B) linear plot of absorbance *vs* concentration (%) at λ_{max} = 270 nm at pH 2 in the Tris buffer for the calibration of isoniazid.

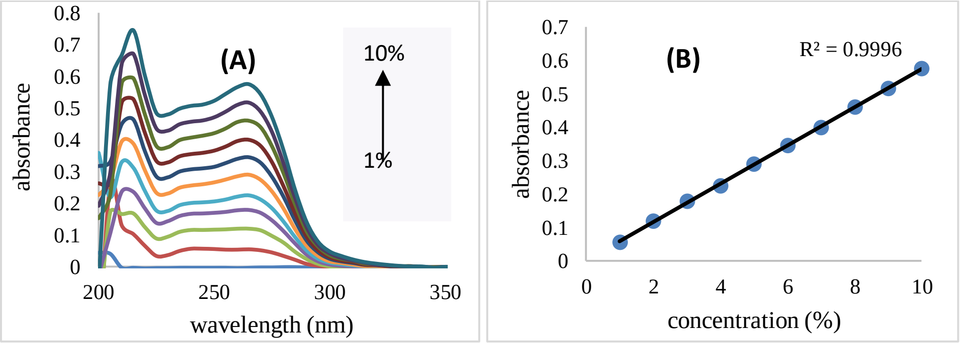

Figure S5. Calibration curve for absorbance of i*n vitro* release of ATDs at pH 8 in the Tris buffer: (A) Absorbance spectra of isoniazid at pH 8; (B) linear plot of absorbance *vs* concentration (%) at λ_{max} = 270 nm at pH 8 in the Tris buffer for the calibration of isoniazid.

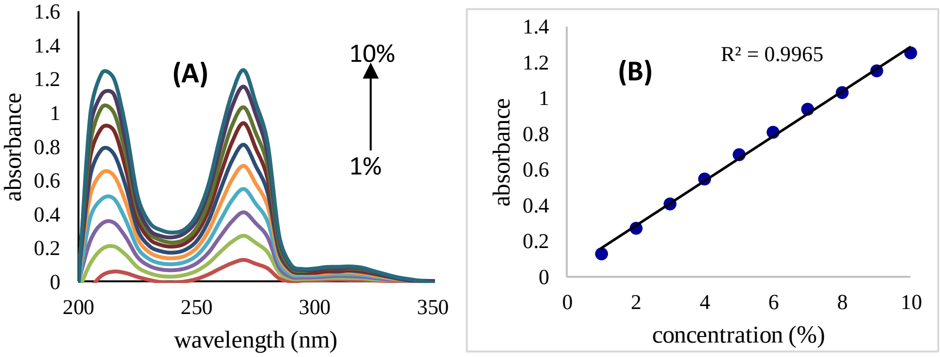

Figure S6. Calibration curve for absorbance of i*n vitro* release of ATDs at pH 2 in the Tris buffer: (A) Absorbance spectra of pyrazinamide at pH 2; (B) linear plot of absorbance *vs* concentration (%) at λ_{max} = 270 nm at pH 2 in the Tris buffer for the calibration of pyrazinamide.

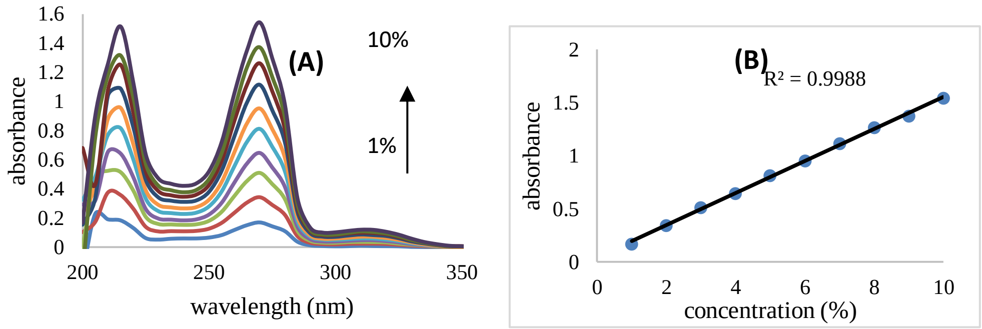

Figure S7. Calibration curve for absorbance of i*n vitro* release of ATDs at pH 8 in the Tris buffer: (A) Absorbance spectra of pyrazinamide at pH 8; (B) linear plot of absorbance *vs* concentration (%) at λ_{max} = 270 nm at pH 8 in the Tris buffer for the calibration of pyrazinamide.

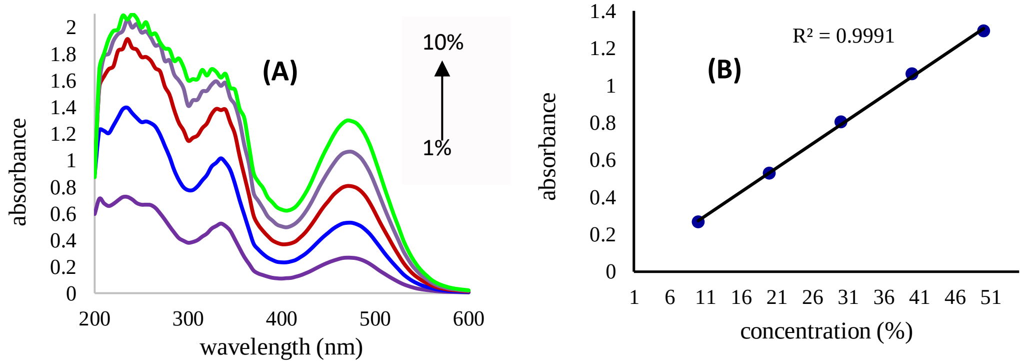

Figure S8. Calibration curve for absorbance of i*n vitro* release of ATDs at pH 2 in the Tris buffer: (A) Absorbance spectra of rifampicin at pH 2; (B) linear plot of absorbance *vs* concentration (%) at λ_{max} = 475 nm for the calibration of rifampicin at pH 2 in the Tris buffer.

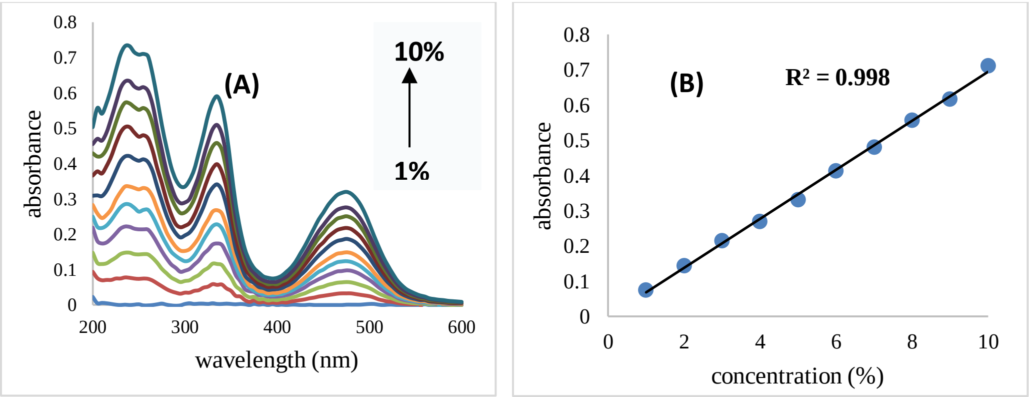

Figure S9. Calibration curve for absorbance of i*n vitro* release of ATDs at pH 8 in the Tris buffer: (A) Absorbance spectra of rifampicin at pH 8; (B) linear plot of absorbance *vs* concentration (%) at λ_{max} = 475 nm for the calibration of rifampicin at pH 8 in the Tris buffer.

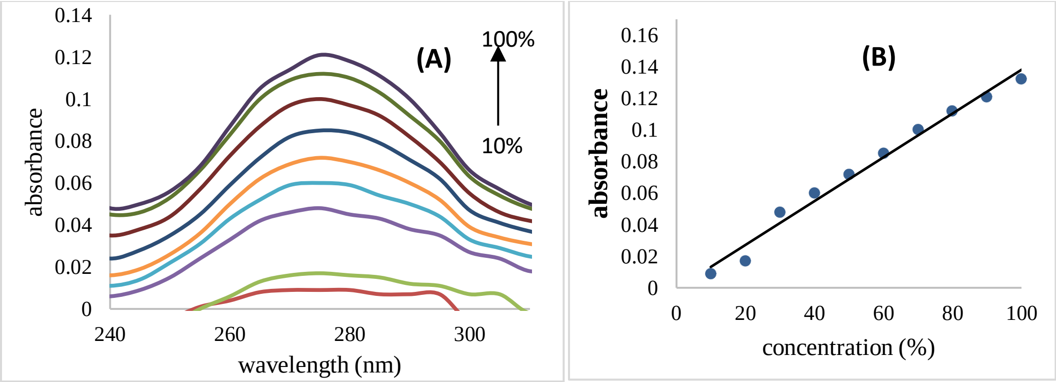

Figure S10. Calibration curve for absorbance of i*n vitro* release of ATDs at pH 2 in the Tris buffer: (A) Absorbance spectra of streptomycin at pH 2; (B) linear plot of absorbance *vs* concentration (%) at λ_{max} = 270 nm for the calibration of streptomycin at pH 2 in the Tris buffer.

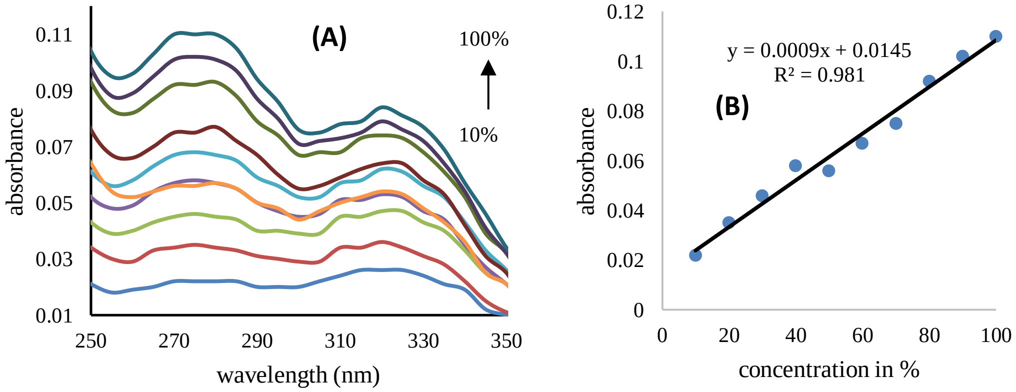

Figure S11. Calibration curve for absorbance of i*n vitro* release of ATDs at pH 8 in the Tris buffer: (A) Absorbance spectra of streptomycin at pH 8; (B) linear plot of absorbance *vs* concentration (%) at λ_{max} = 280 nm for the calibration of streptomycin at pH 8 in the Tris buffer.

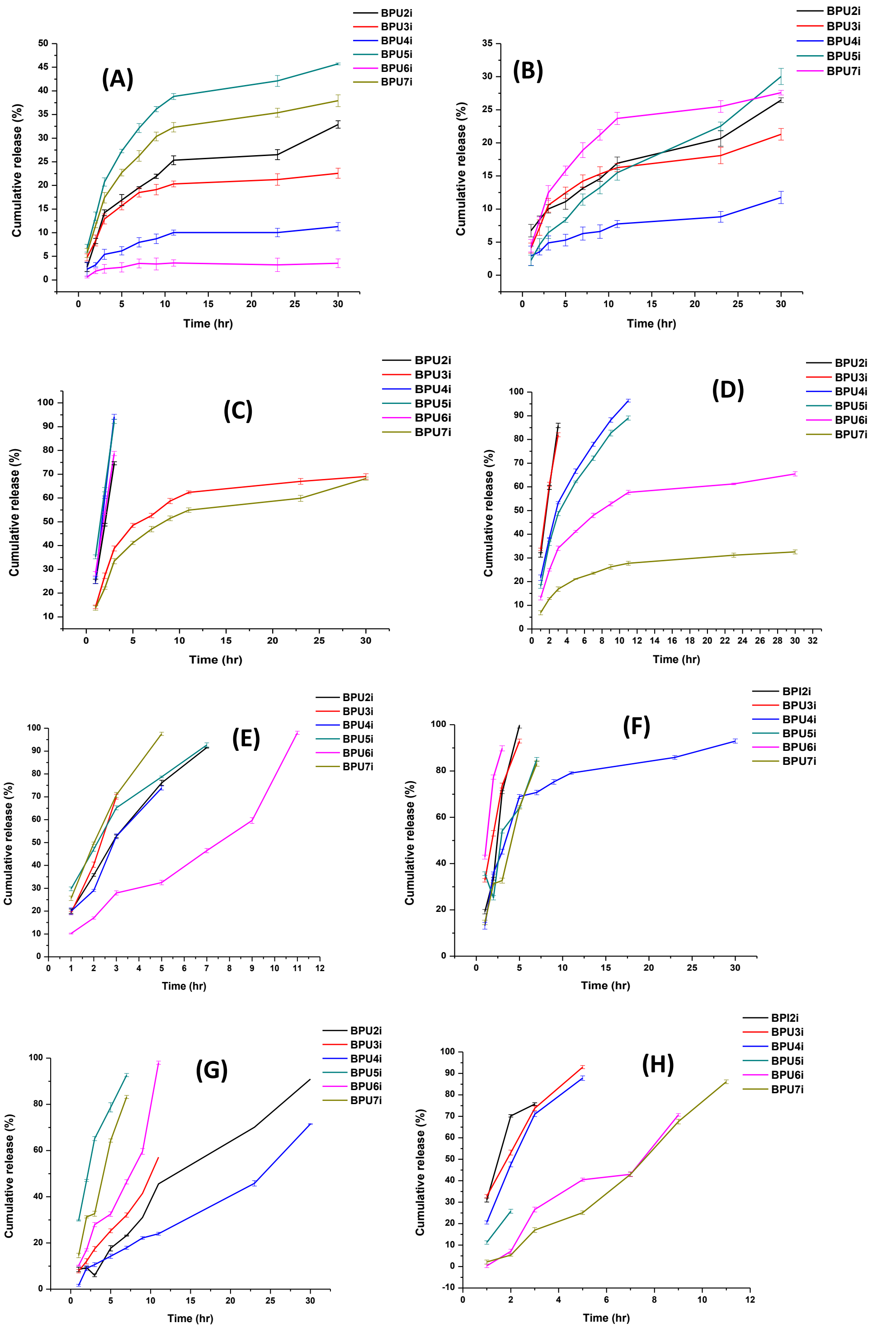

Figure S12. Plotting of cumulative release percentage (CR%) of ATDs-loaded bulk-polyurethanes (SBPU**2i**-**7i**) *vs* time (h) in the Tris buffer at different pH: (A) pyrazinamide-loaded bulk-polyurethanes (SBPU**2i**-**7i**) at pH 2; (B) pyrazinamide-loaded bulk-polyurethanes (SBPU**2i**-**7i**) at pH 8; (C) isoniazid-loaded bulk-polyurethanes (SBPU**2i**-**7i**) at pH 2; (D) isoniazid-loaded bulk-polyurethanes (SBPU**2i**-**7i**) at pH 8; (E) rifampicin-loaded bulk-polyurethanes (SBPU**2i**-**7i**) at pH 2; (F) rifampicin-loaded bulk-polyurethanes (SBPU**2i**-**7i**) at pH 8; (G) streptomycin-loaded bulk-polyurethanes (SBPU**2i**-**7i**) at pH 2; (H) streptomycin-loaded bulk-polyurethanes (SBPU**2i**-**7i**) at pH 8.

Method for Minimal Inhibition Concentration: Lowenstein–Jensen (LJ) slope method for *in vitro* anti-tuberculosis efficacy

METHODS USED FOR SCREENING: The individual drug was diluted to 1000.0 µg/mL concentration as a stock solution for *in vitro* study and not for cell lines. Sample (1.0 mg), solvent (1.0 mL) and sterile D/W were mixed to get the final concentration as 1000.0 µg/mL. This solution was diluted by serial dilution method. 2% DMSO, which is safe for bacteria, was used as per CLSI guidelines. For this study, LJ medium was prepared as mentioned below.

Preparation of Lowenstein-Jensen medium:

Ingredients for the preparation of Lowenstein-Jensen medium:	
**Mineral salt and reagents**	**Weight**
Potassium dihydrogen phosphate anhydrous (KH_2PO_4)	2.4 g
Magnesium sulphate anhydrous	0.24 g
Magnesium citrate	0.6 g
Asparagine	3.6 g
Glycerol	12.0 mL
Malachite green, 2% solution*	20.0 mL
Distilled water	600.0 mL
*Malachite green solution 2% was prepared by mixing of malachite green dye (2.0 g) powder made with a mortar and pestle in 100.0 mL distilled water.	

Homogenized eggs solution:

Hens' fresh eggs were washed with the soap solution and rinsed with water and allowed to dry. Then the egg was broken into a sterile flask. 620.0 mL mineral salt solution with malachite green and 1000.0 mL homogenized eggs (25-30) were mixed, and the mixture was battered with a sterile blender.

The 6.0-8.0 mL of egg medium was taken into each sterile McCartney bottle and the caps were tightly closed and made inspissated without delay by placing the container in a slanted position in the inspissator and coagulate the medium for 30 min at 85 °C. After overnight, it was re-inspissated for 30 min and then it was stored at room temperature.

Sterility check and storage:

After inspissation, the whole media batch or a representative sample of the media bottles was incubated at 37˚C for 24 hours.

Screening process: For screening purposes, 100.0, 50.0, 12.5, 6.25. 3.125, 10.0, 5.0, 2.5, 1.25, 8.0, 4.0, 2.0, 1.0, 0.5, 0.25 µg/mL concentrations of the synthesized drugs were considered. Then LJ medium was added. The active synthesized drugs found in this primary screening were further tested in the second set of dilution against strains.

Reading result:

MIC was measured from the highest dilution showing at least 99% inhibition, which is greatly affected by the size of the inoculum. It was realized that the test mixture required 10^8 organisms/mL, and the result was compared with the McFarland standard. The experiment revealed the visual growth on L.J media, and it was observed that the lowest dilution of the drug had no growth on media with positive control.

The standard drugs:

The standard strain *M. tuberculosis*, H37Rv was evaluated with each new batch of medium. The recommended drug concentrations were 4 mg/L for streptomycin, 0.2 mg/L for isoniazid, 40.0 mg/L for rifampicin.

Spectroscopic data:

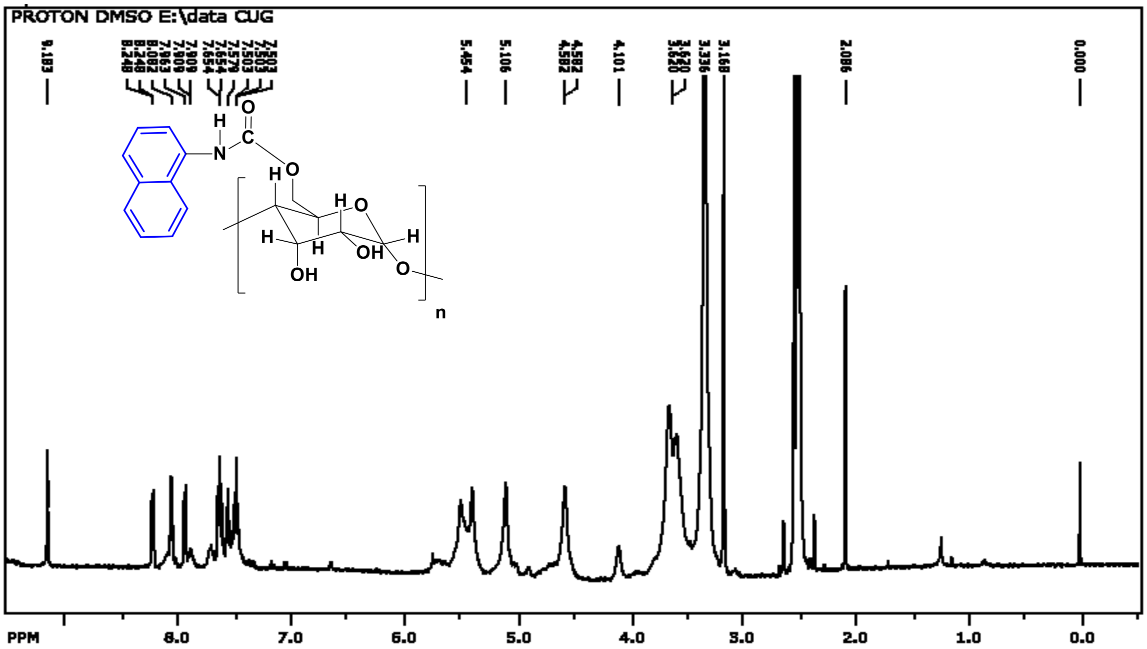

Figure S13. ^{1}H NMR spectra of SBPU**5i** in DMSO-d6 at 500 MHz

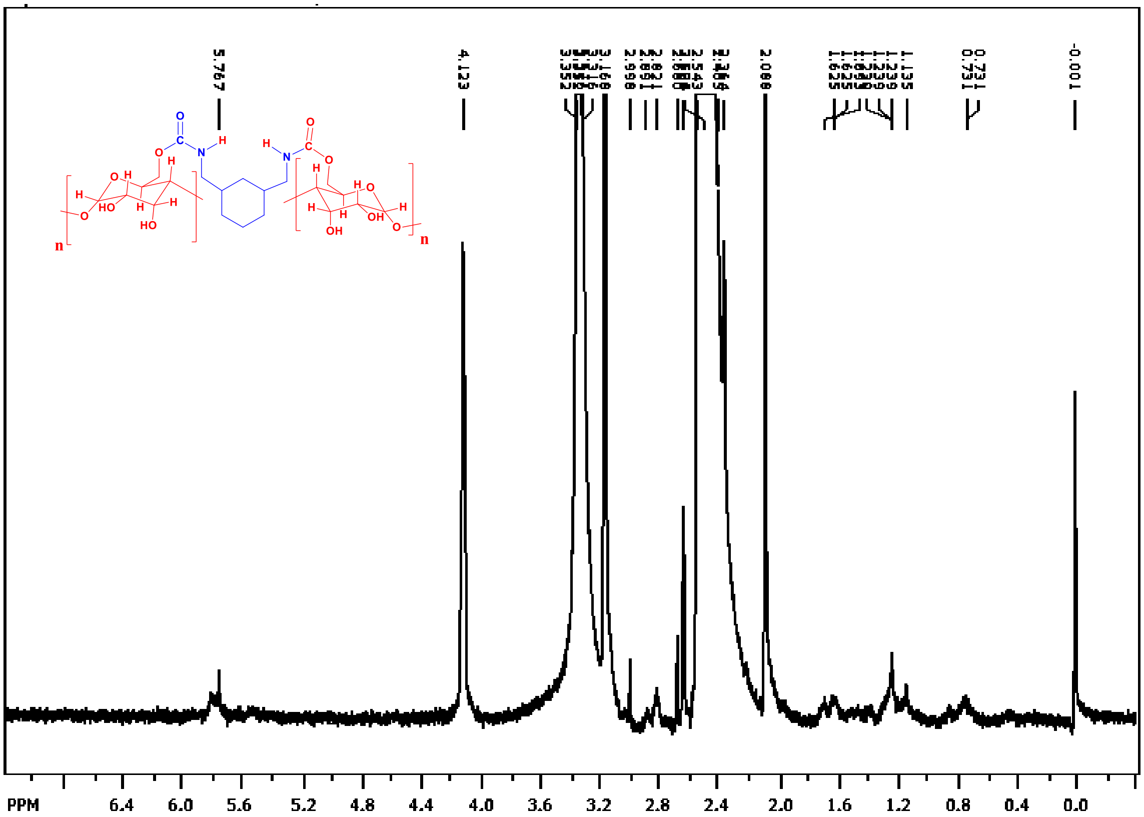

Figure S14. ^{1}H NMR spectra of SBPU**6i** in DMSO-d6 at 500MHz

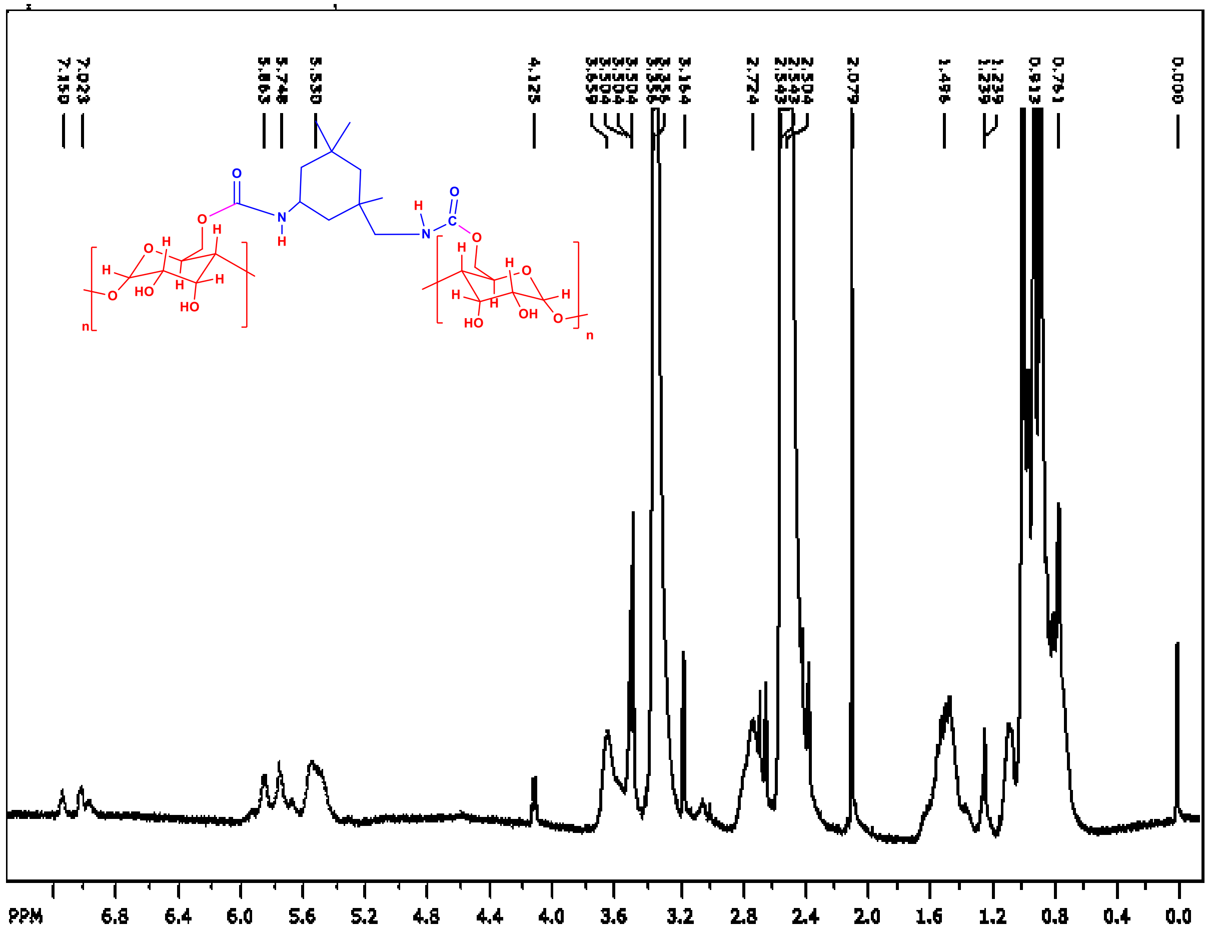

Figure S15. ^{1}H NMR spectra of SBPU**7i** in DMSO-d6 at 500MHz

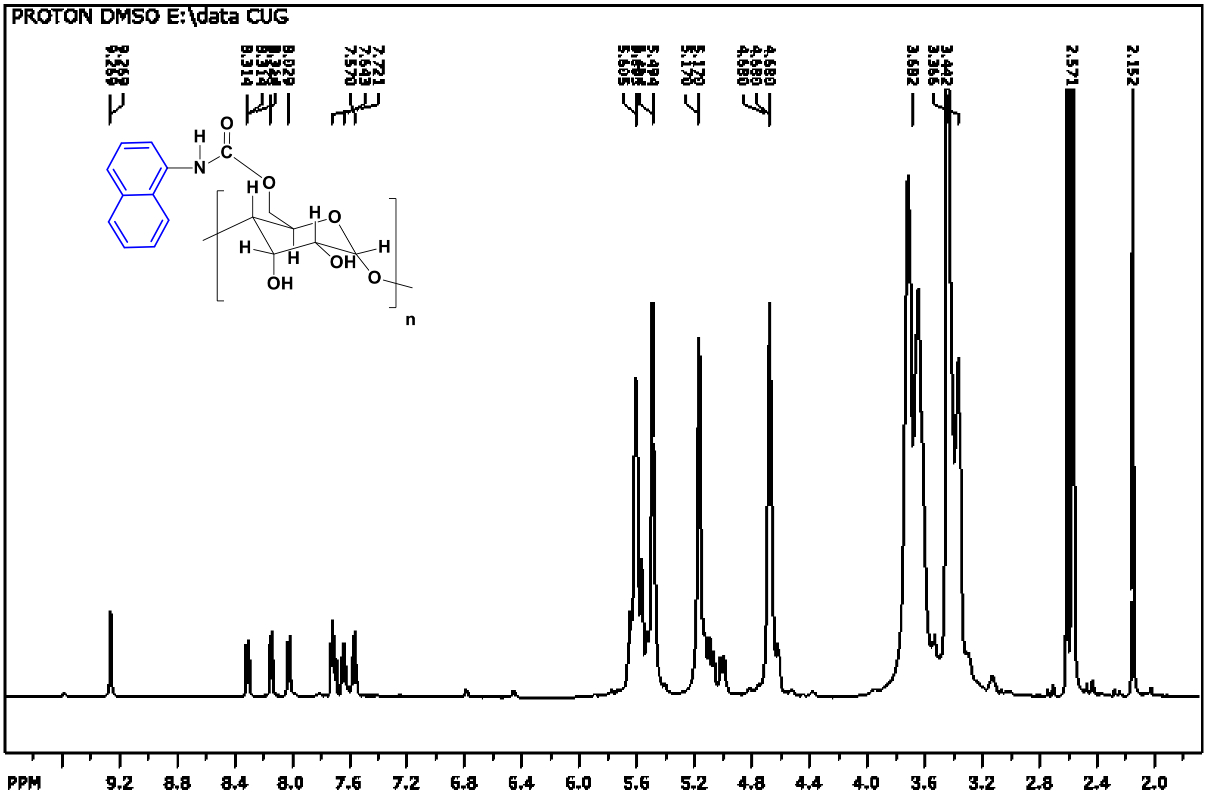

Figure S16. ^{1}H NMR spectra of SNPU**5i** in DMSO-d6 at 500MHz

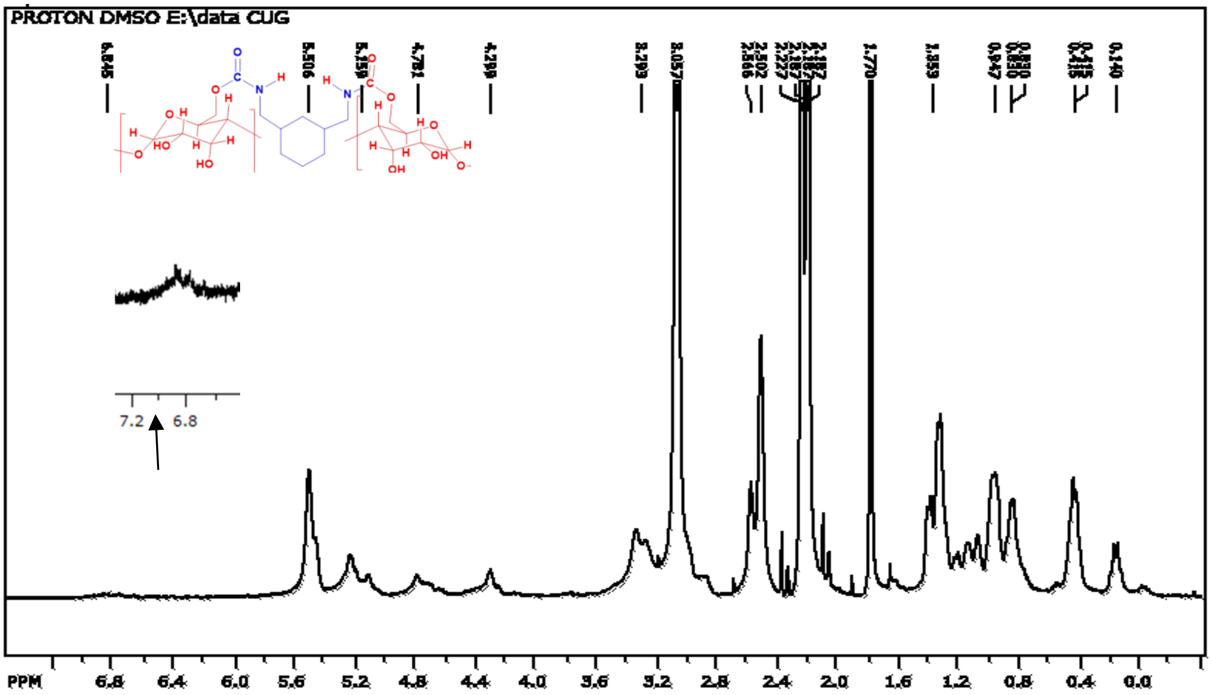

Figure S17. ^{1}H NMR spectra of SNPU**6i** in DMSO-d6 at 500MHz

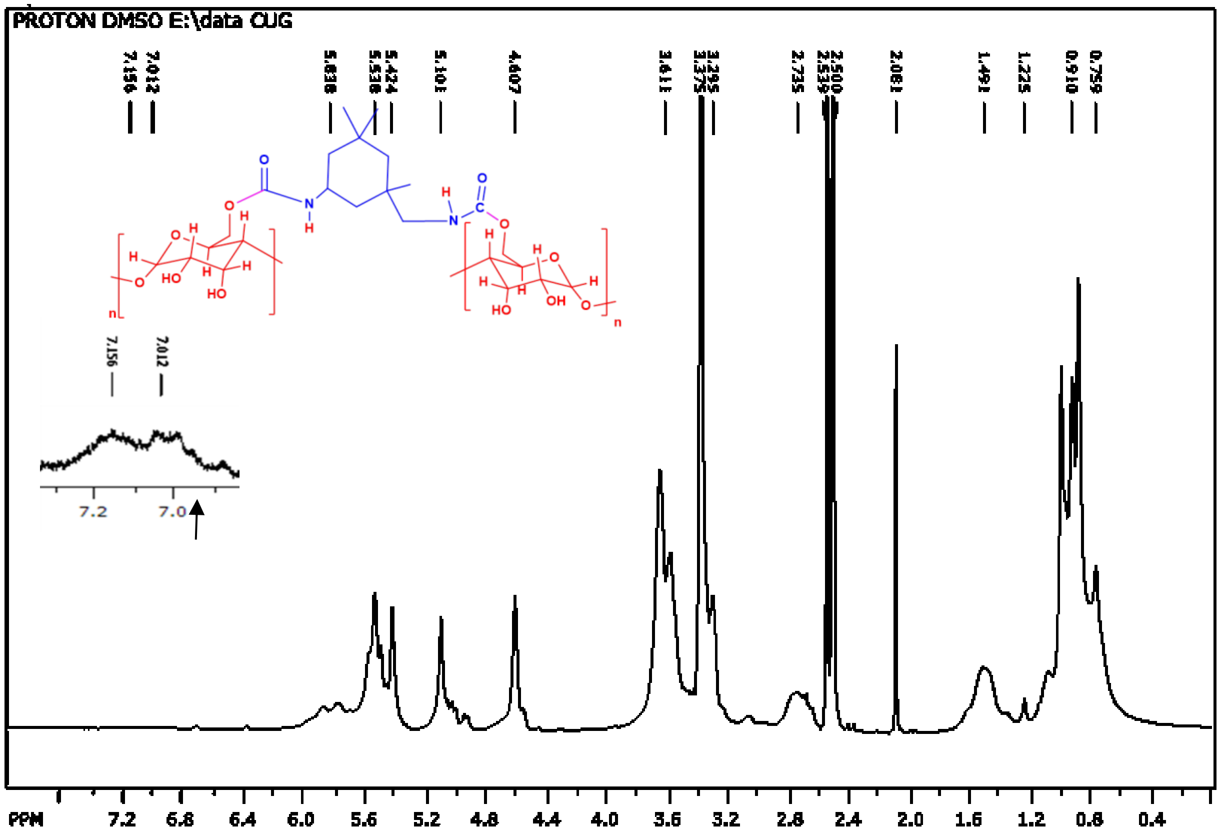

Figure S18. ^{1}H NMR spectra of SNPU**7i** in DMSO-d6 at 500MHz

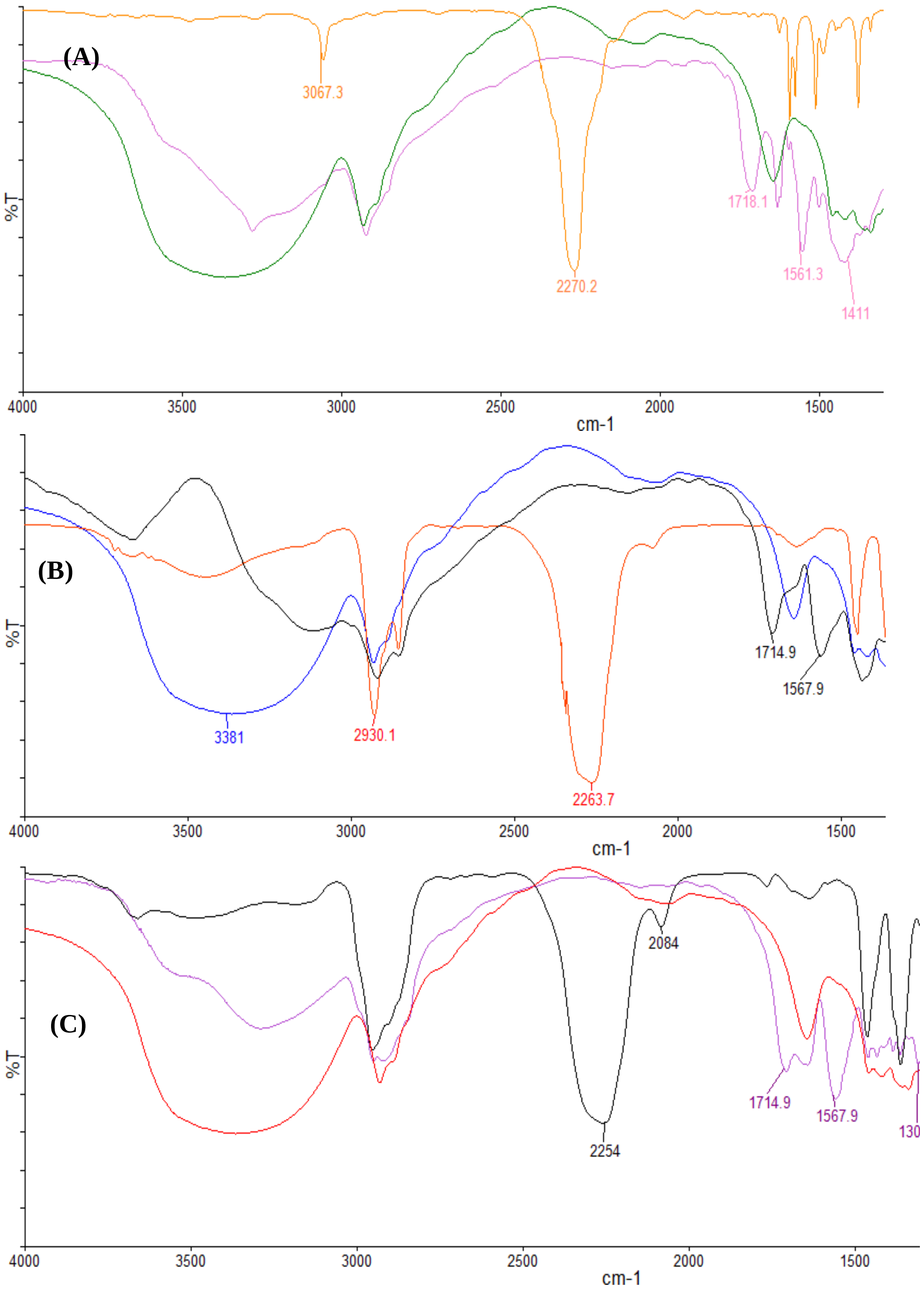

Figure S19. FTIR spectra of SBPU**5i-7i (A-C)**

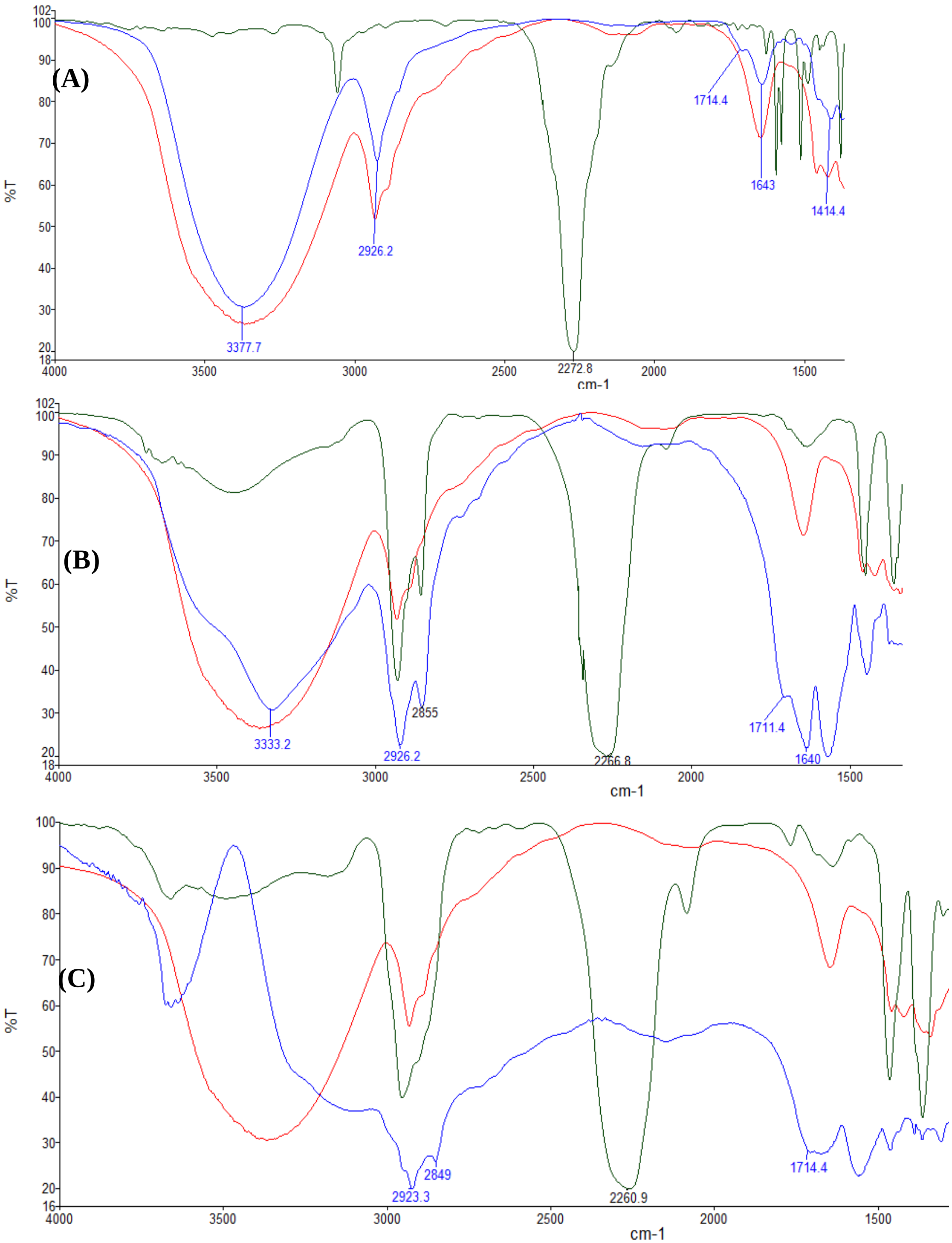

Figure S20. FTIR spectra of SNC compared with the FTIR spectra of respective isocyanate (**5i-7i**) and SNPU**5i-7i (A-C)**

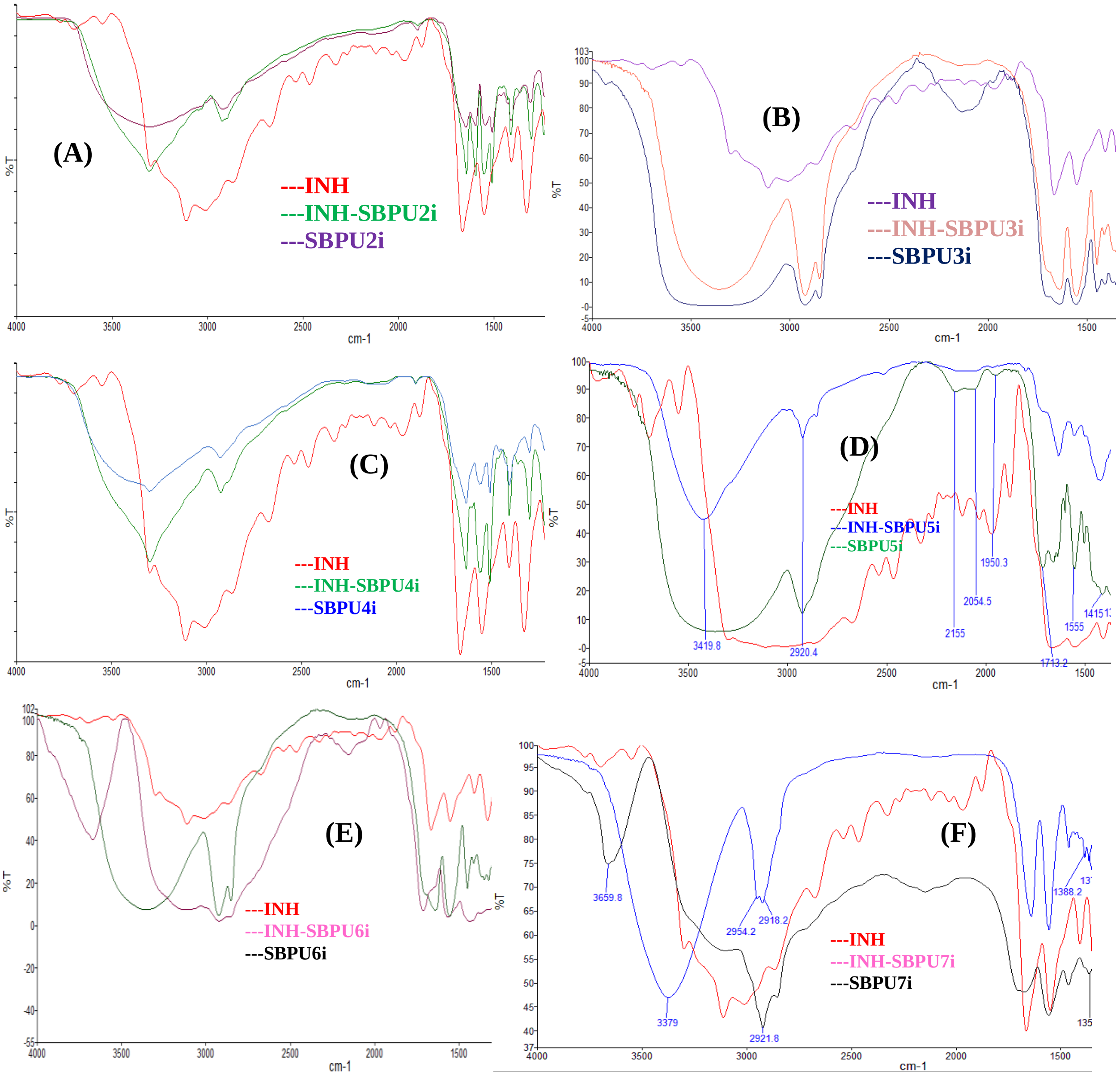

Figure S21. FTIR Spectra of ATDs-loaded bulk polyurethanes: Comparison of FTIR spectra of isoniazid (INH) with that of **(A)** INH-SBPU**2i** and **SBPU2i;** (**B**) INH-SBPU**3i** and SBPU**3i**; **(C)** INH-SBPU**4i** and **SBPU4i; (D)** INH-SBPU**5i** and SBPU**5i; (E)** INH-SBPU**6i** and **SBPU6i;** (**F**) INH-SBPU**7i** and **SBPU7i**

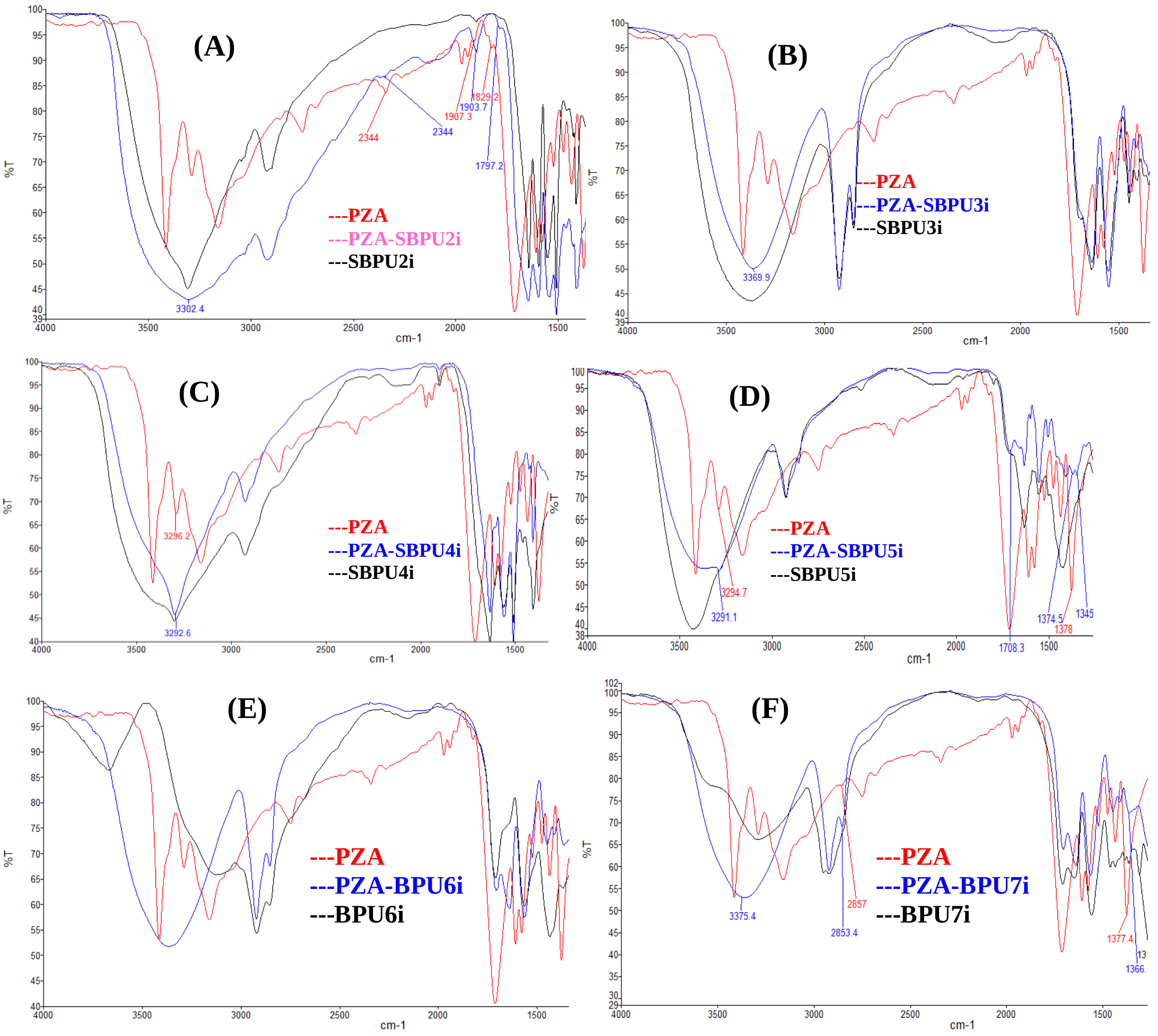

Figure S22. FTIR Spectra of ATDs-loaded bulk polyurethanes: Comparison of FTIR spectra of isoniazid (PZA) with that of (A) PZA-SBPU**2i** and **S**BPU**2i;** (B) PZA-SBPU**3i** and SBPU**3i**; (C) PZA-SBPU**4i** and **S**BPU**4i;** (D) PZA-SBPU**5i** and SBPU**5i;** (E) PZA-SBPU**6i** and **S**BPU**6i;** (F) PZA-SBPU**7i** and **S**BPU**7i**

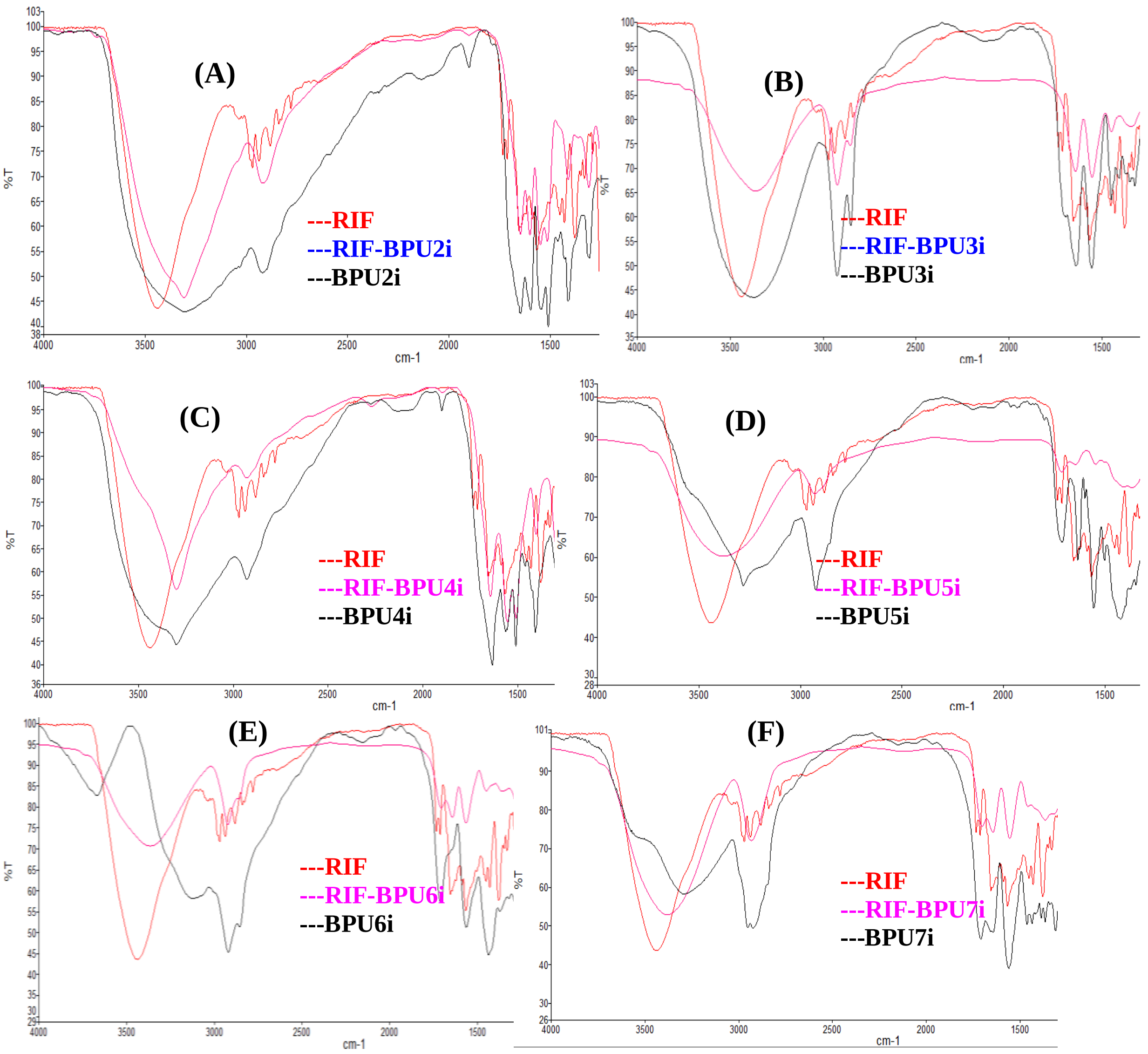

Figure S23. FTIR Spectra of ATDs-loaded bulk polyurethanes: Comparison of FTIR spectra of rifampicin (RIF) with that of (A) RIF-SBPU**2i**, **S**BPU**2i;** (B) RIF-SBPU**3i** and SBPU**3i**; (C) RIF-SBPU**4i** and **S**BPU**4i;** (D) RIF-SBPU**5i** and SBPU**5i;** (E) RIF-SBPU**6i** and **S**BPU**6i;** (F) RIF-SBPU**7i** and **S**BPU**7i**

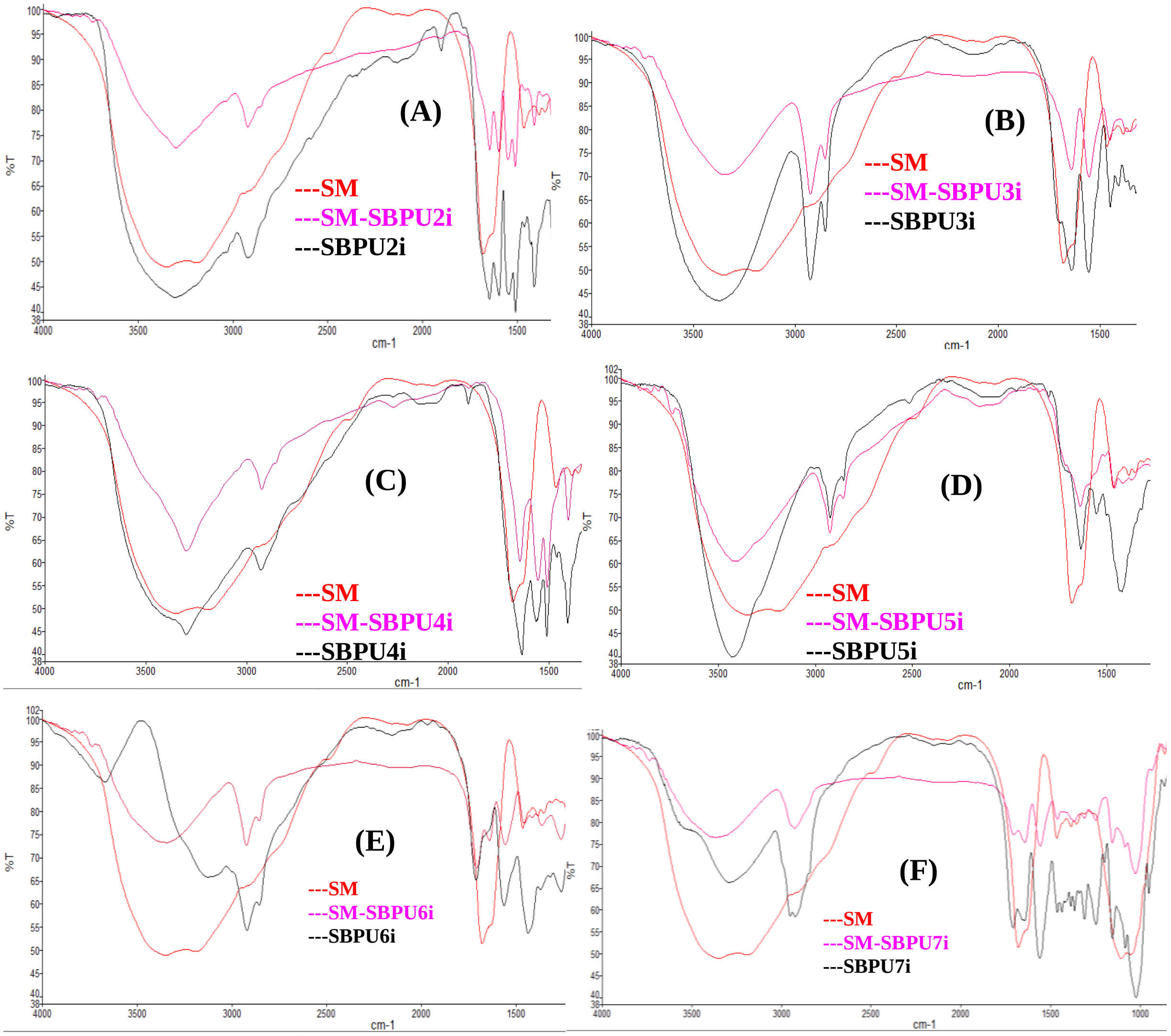

Figure S24. FTIR Spectra of ATDs-loaded bulk polyurethanes: Comparison of FTIR spectra of streptomycin (SM) with that of (A) SM-SBPU**2i** and SBPU**2i;** (B) SM-SNPU**3i** and SBPU**3i**; (C) SM-SBPU**4i** and **SBPU4i;** (D) SM-SBPU**5i** and SBPU**5i;** (E) SM-SBPU**6i** and **S**NPU**6i;** (F) SM-SBPU**7i** and **S**BPU**7i**

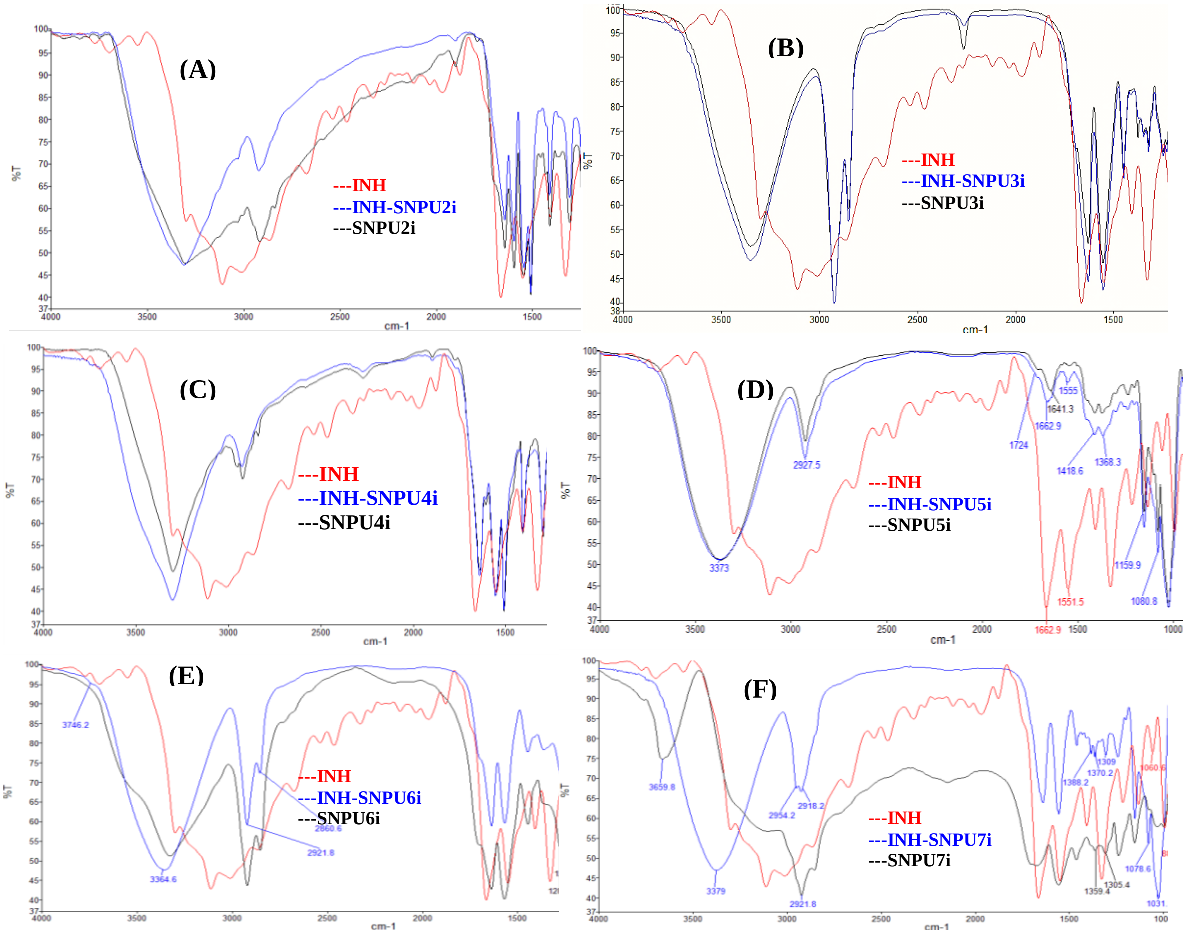

Figure S25. FTIR Spectra of ATDs-loaded starch nanopolyurethanes: Comparison of FTIR spectra of isoniazid (INH) with that of (A) INH-SNPU**2i** and SNPU**2i;** (B) INH-SNPU**3i** and SNPU**3i**; (C) INH-SNPU**4i** and **S**NPU**4i;** (D) INH-SNPU**5i** and SNPU**5i;** (E) INH-SNPU**6i** and **S**NPU**6i;** (F) INH-SNPU**7i** and **S**NPU**7i**

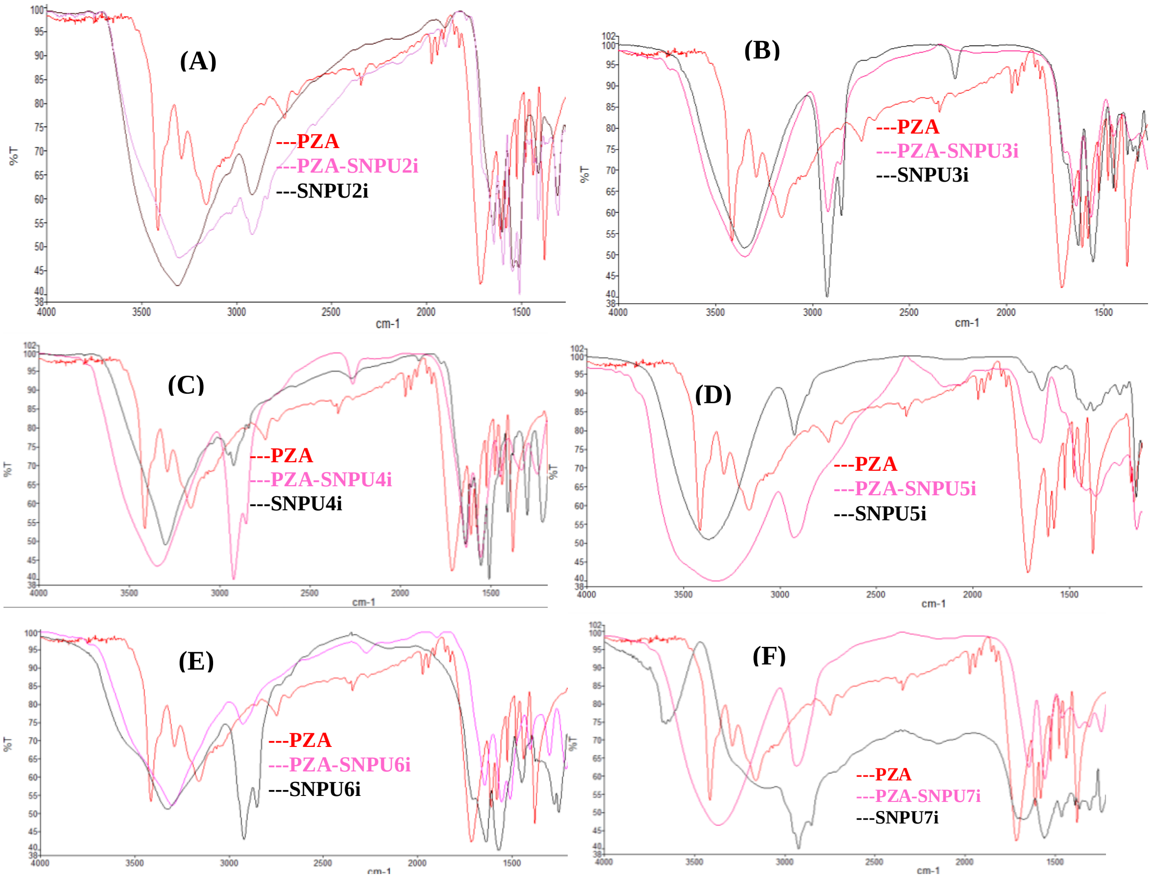

Figure S26. FTIR Spectra of ATDs-loaded starch nanopolyurethanes: Comparison of FT-IR spectra of pyrazinamide (PZA) with that of (A) PZA-SNPU**2i** and SNPU**2i;** (B) PZA-SNPU**3i** and SNPU**3i**; (C) PZA -SNPU**4i** and **S**NPU**4i;** (D) PZA-SNPU**5i** and SNPU**5i;** (E) PZA-SNPU**6i** and SNPU**6i;** (F) PZA-SNPU**7i** and **S**NPU**7i**

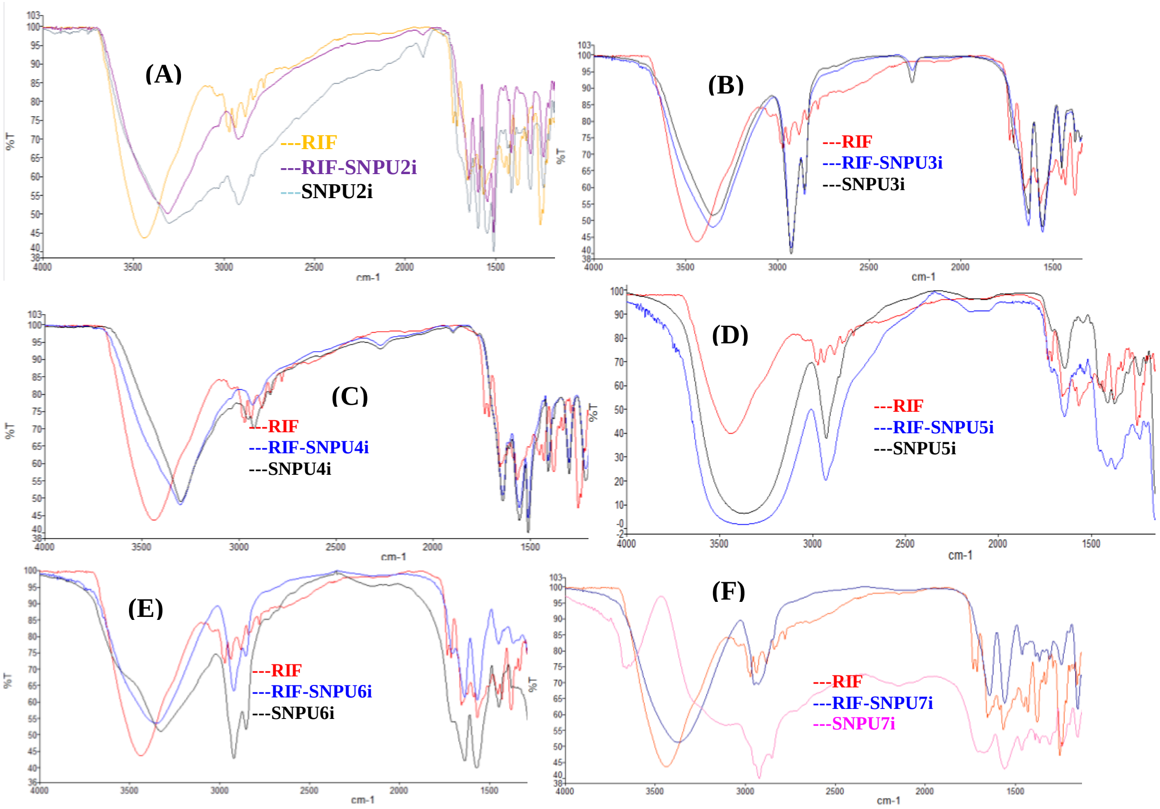

Figure S27. FTIR Spectra of ATDs-loaded starch nanopolyurethanes: Comparison of FT-IR spectra of rifampicin (RIF), with that of (A) RIF-SNPU**2i** and SNPU**2i;** (B) RIF-SNPU**3i** and SNPU**3i**; (C) RIF-SNPU**4i** and **S**NPU**4i;** (D) RIF-SNPU**5i** and SNPU**5i;** (E) RIF -SNPU**6i** and **S**NPU**6i;** (F) RIF-SNPU**7i** and **S**NPU**7i**

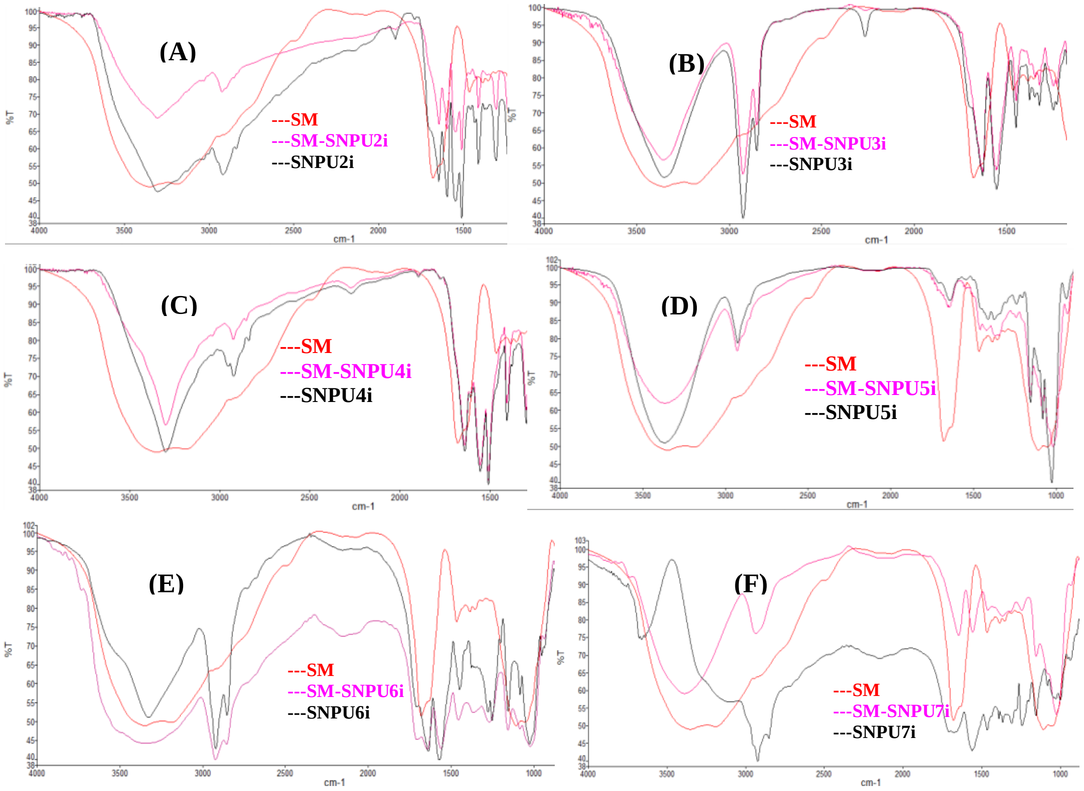

Figure S28. FTIR Spectra of ATDs-loaded starch nanopolyurethanes: Comparison of FT-IR spectra of streptomycin (SM), with that of (A) SM-SNPU2**i,** and SNPU2**i;** (B) SM-SNPU3**i** and SNPU3**i**; (C) SM -SNPU4**i** and **SNPU4i;** (D) SM-SNPU5**i** and SNPU5**i;** (E) SM-SNPU**6i** and **SNPU6i;** (F) SM-SNPU**7i** and **SNPU7i**

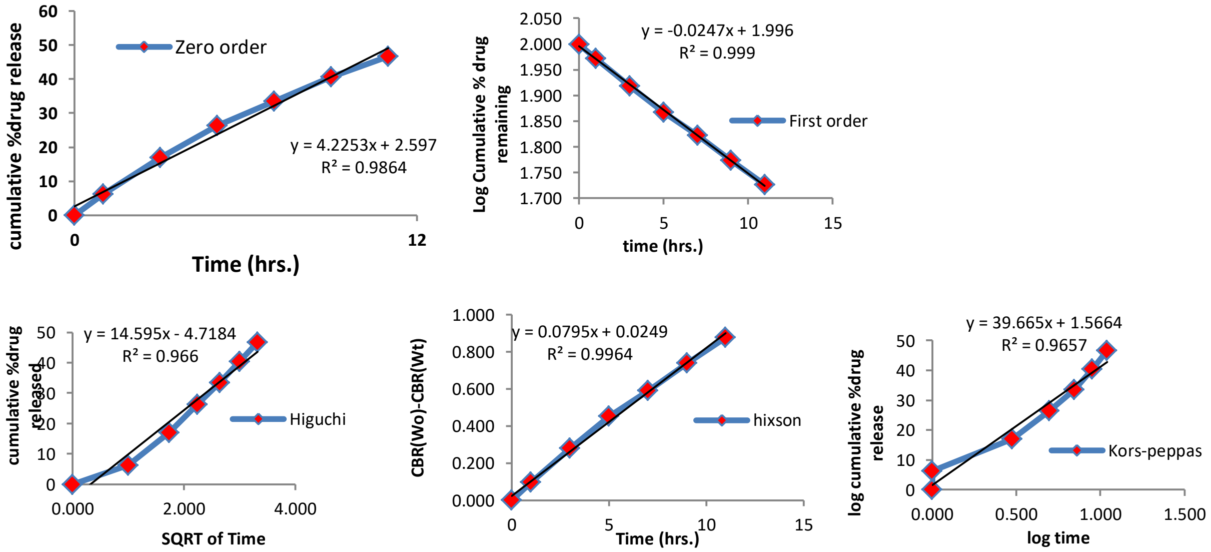

Figure S29 The graphs of Zero order, First order, Higuchi, Hixson-Crowell and Kors-peppas models are plotted for rifampicin (RIF) release kinetics from NPU2i-pH8

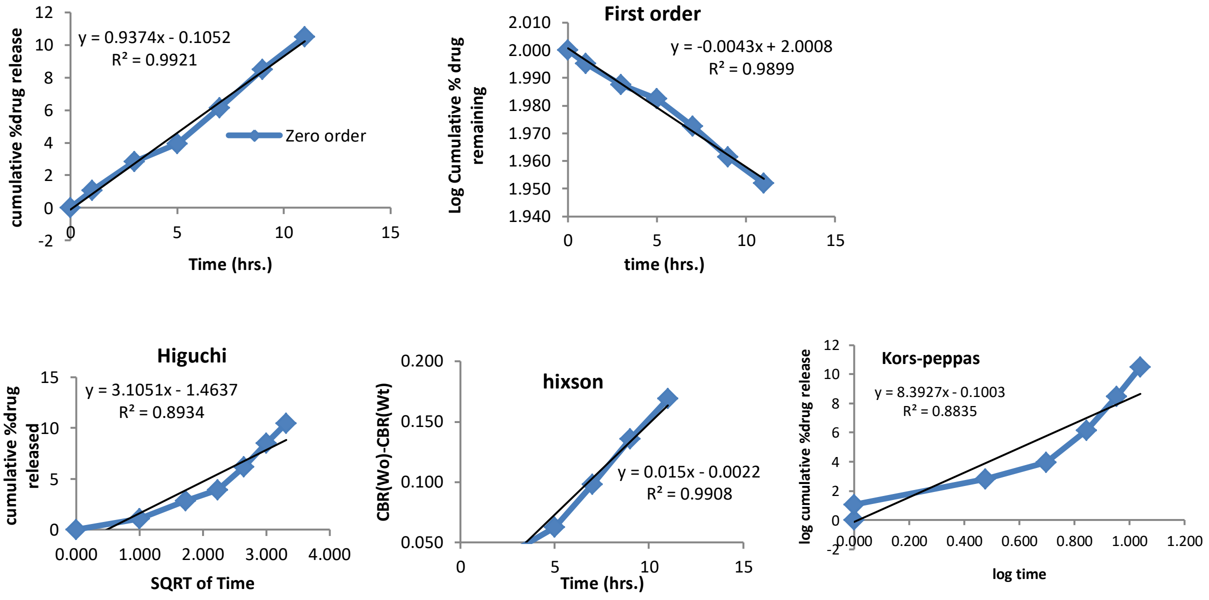

Figure S30 The graphs of Zero order, First order, Higuchi, Hixson-Crowell and Kors-peppas models are plotted for rifampicin (RIF) release kinetics from NPU3i-pH8

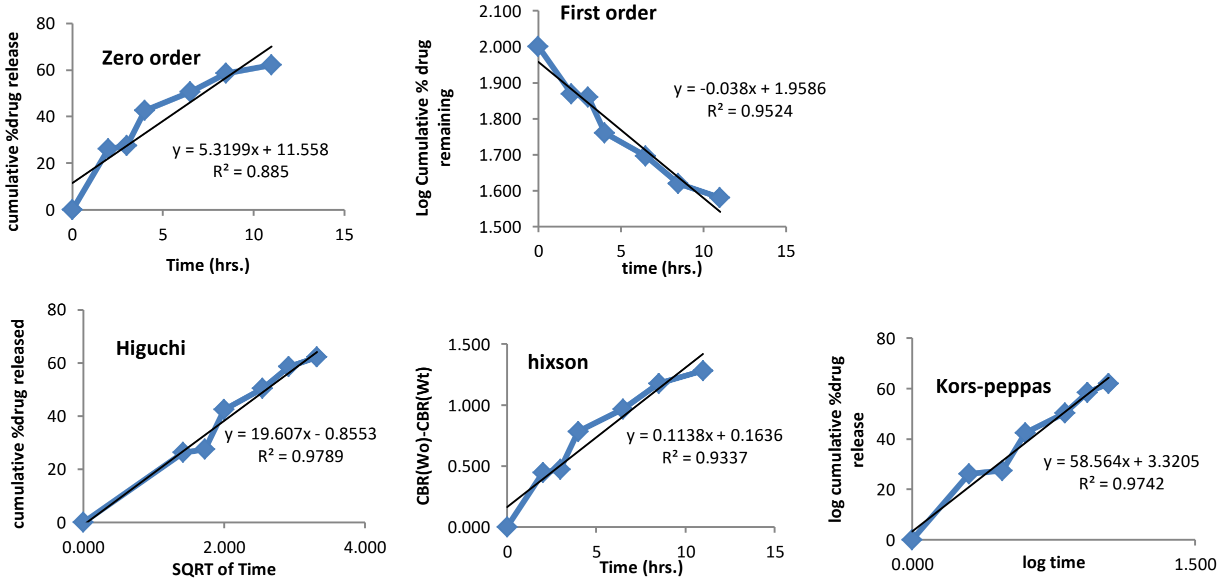

Figure S31 The graphs of Zero order, First order, Higuchi, Hixson-Crowell and Kors-peppas models are plotted for streptomycin (SM) release kinetics from NPU3i-pH8

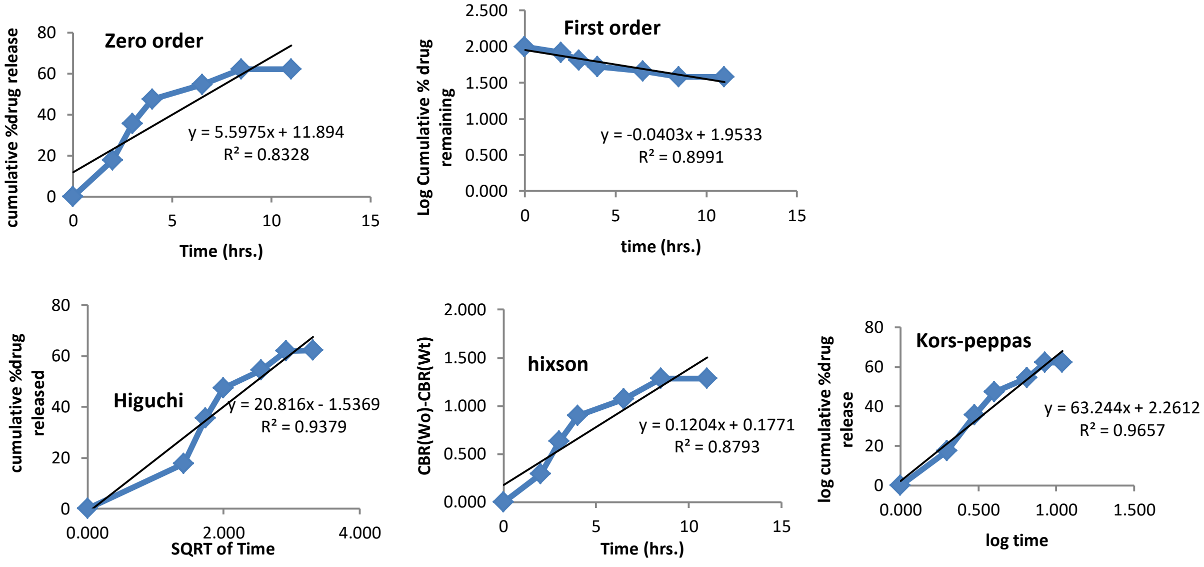

Figure S32 The graphs of Zero order, First order, Higuchi, Hixson-Crowell and Kors-peppas models are plotted for isoniazid (INH) release kinetics from NPU2i-pH2

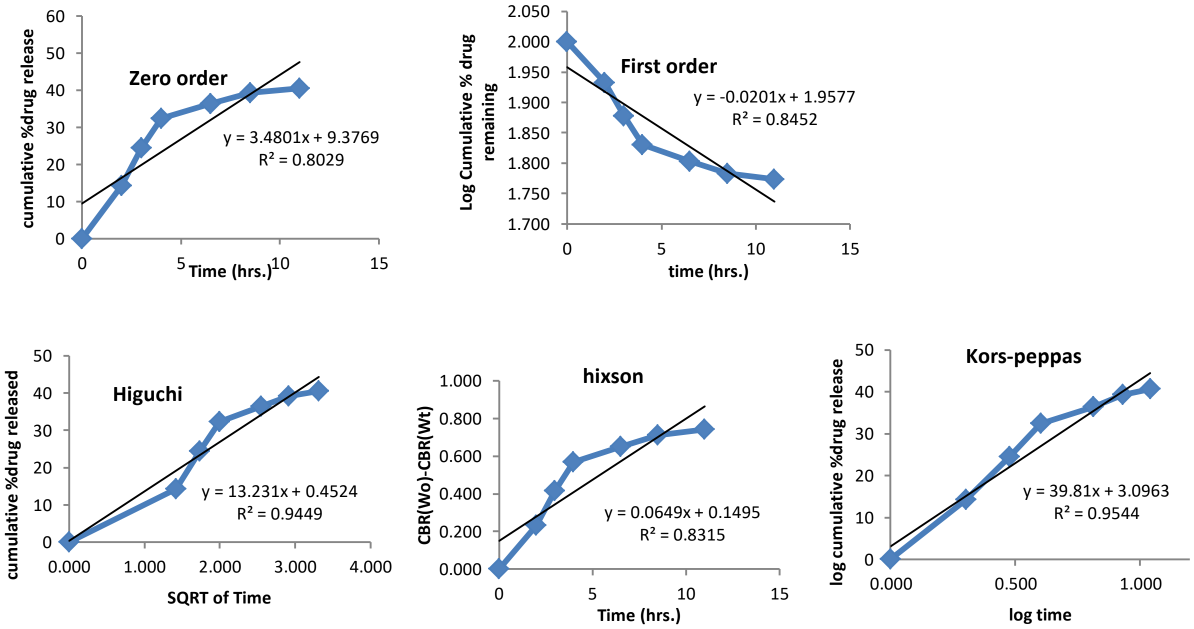

Figure S33 The graphs of Zero order, First order, Higuchi, Hixson-Crowell and Kors-peppas models are plotted for isoniazid (INH) release kinetics from NPU3i-pH2

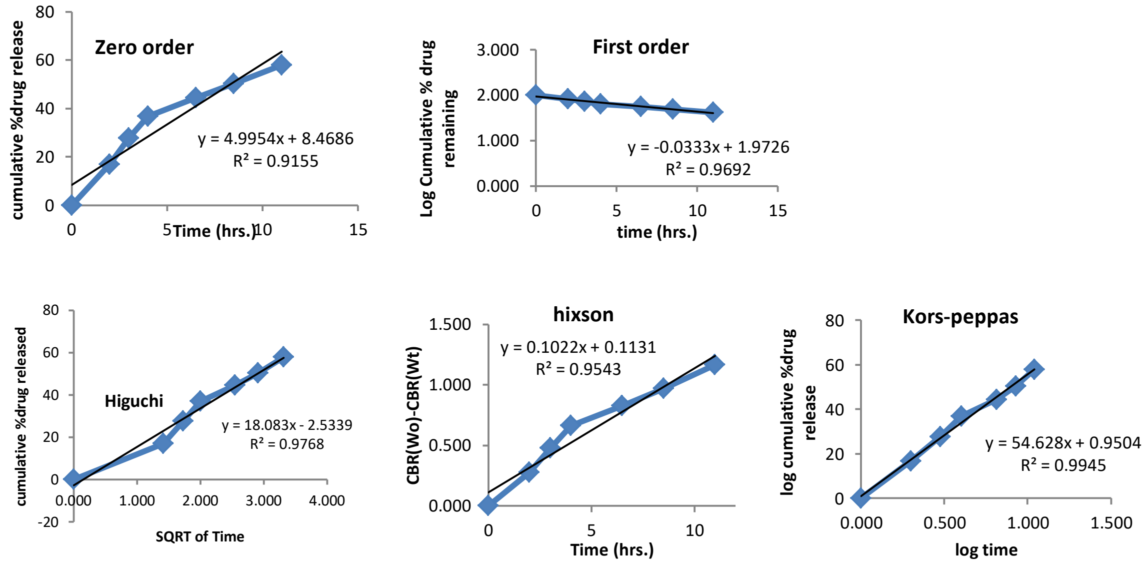

Figure S34 The graphs of Zero order, First order, Higuchi, Hixson-Crowell and Kors-peppas models are plotted for isoniazid (INH) release kinetics from NPU4i-pH2

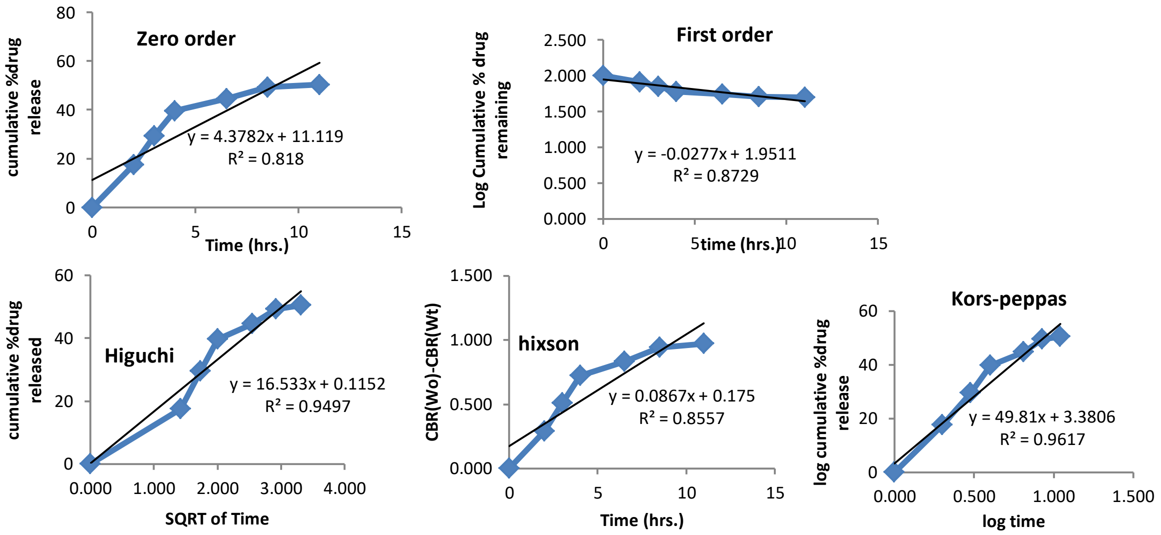

Figure S35 The graphs of Zero order, First order, Higuchi, Hixson-Crowell and Kors-peppas models are plotted for isoniazid (INH) release kinetics from NPU3i-pH8

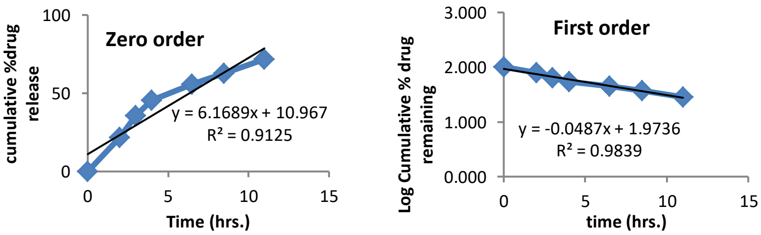

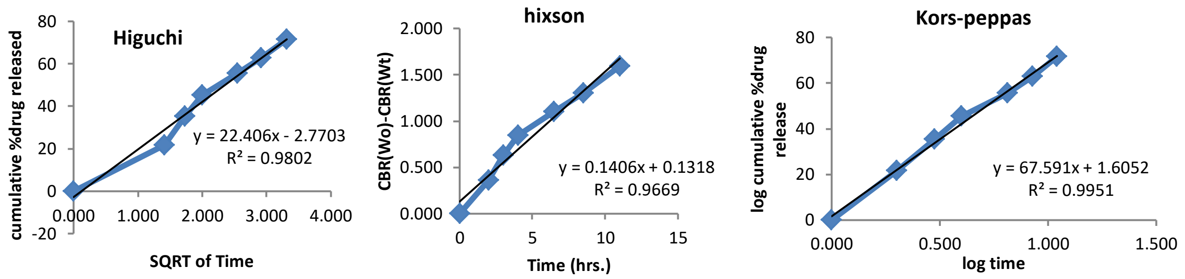

Figure S36 The graphs of Zero order, First order, Higuchi, Hixson-Crowell and Kors-peppas models are plotted for isoniazid (INH) release kinetics from NPU4i-pH8

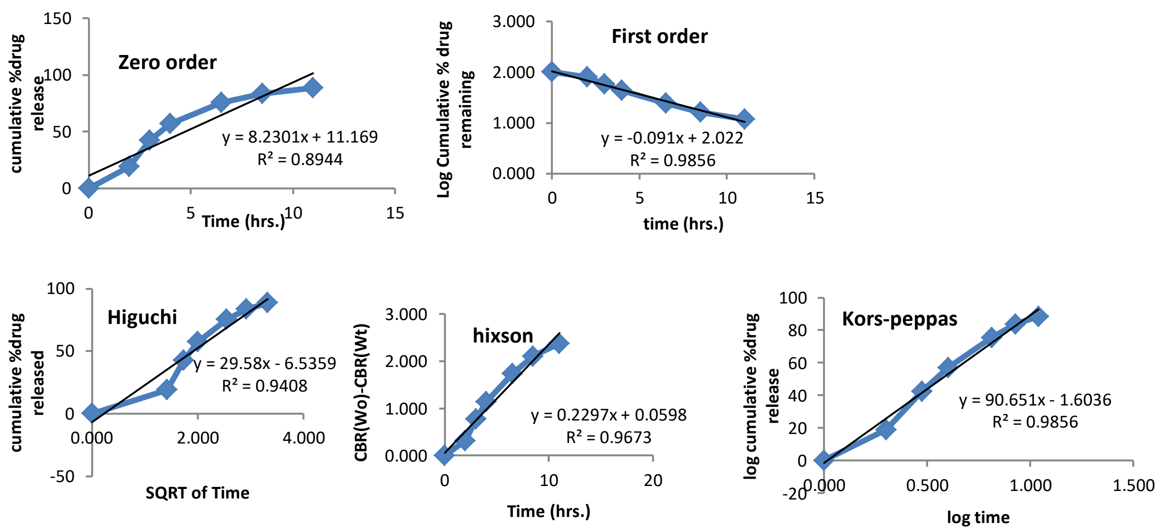

Figure S37 The graphs of Zero order, First order, Higuchi, Hixson-Crowell and Kors-peppas models are plotted for pyrazinamide (PZA) release kinetics from NPU5i-pH2

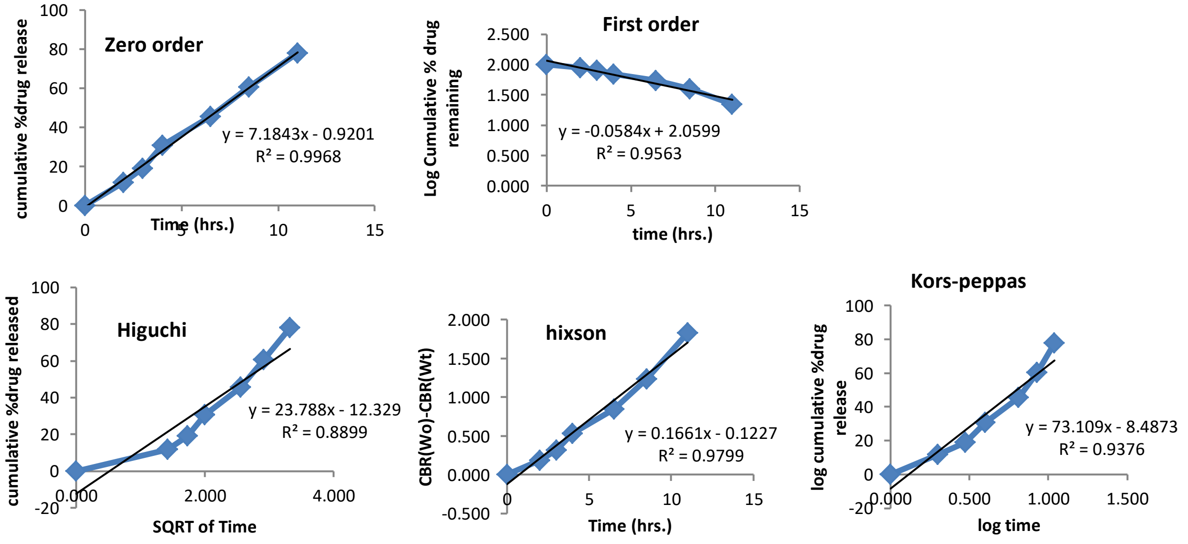

Figure S38 The graphs of Zero order, First order, Higuchi, Hixson-Crowell and Kors-peppas models are plotted for pyrazinamide (PZA) release kinetics from NPU2i-pH8

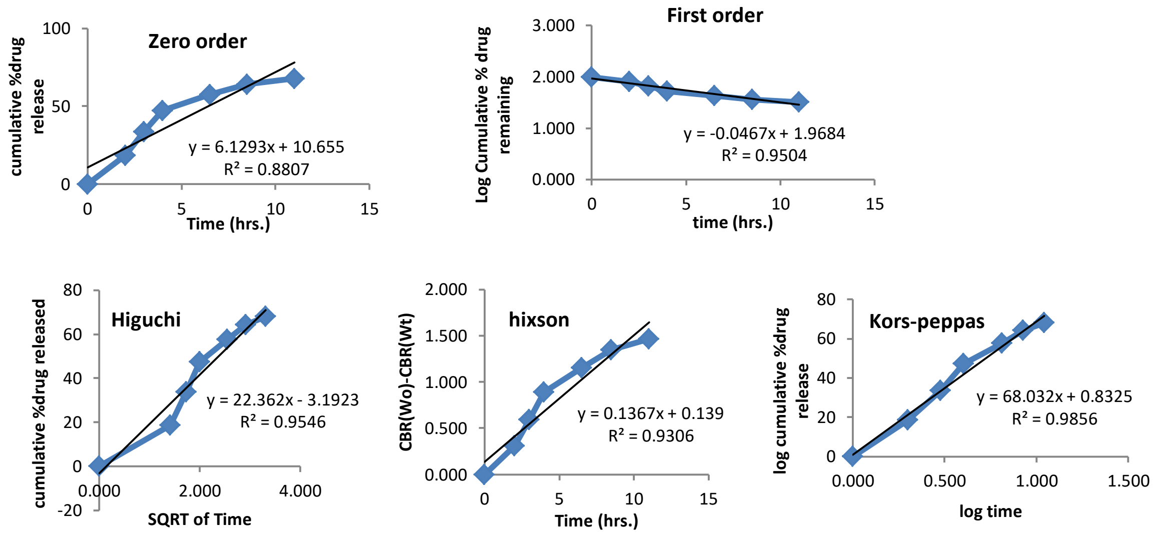

Figure S39 The graphs of Zero order, First order, Higuchi, Hixson-Crowell and Kors-peppas models are plotted for pyrazinamide (PZA) release kinetics from NPU5i-pH8

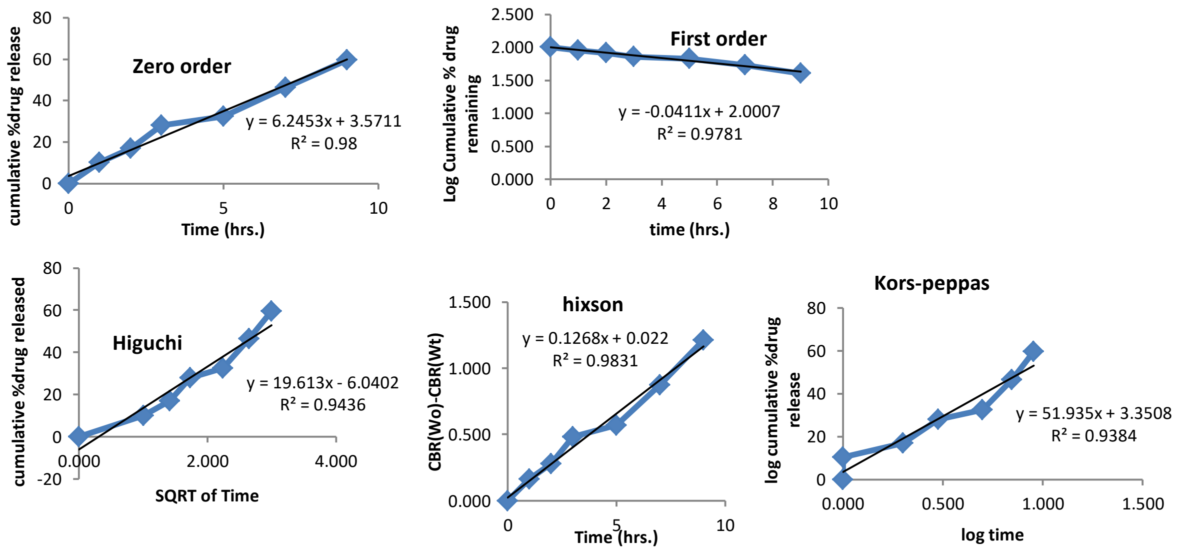

Figure S40 The graphs of Zero order, First order, Higuchi, Hixson-Crowell and Kors-peppas models are plotted for rifampicin (RIF) release kinetics from BPU6i-pH2

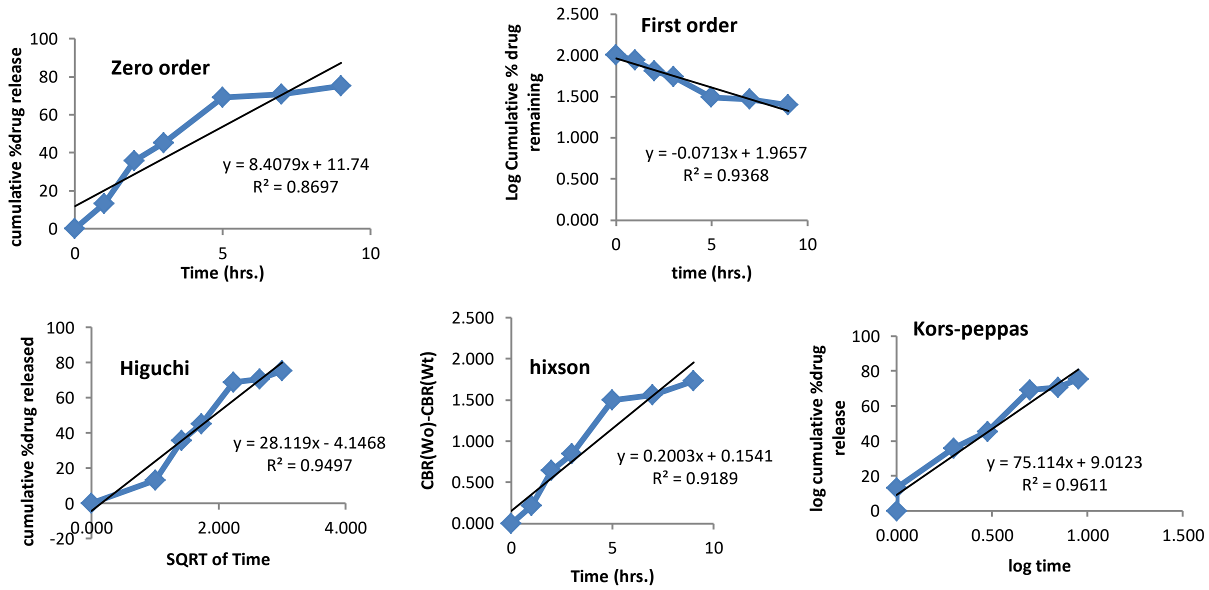

Figure S41 The graphs of Zero order, First order, Higuchi, Hixson-Crowell and Kors-peppas models are plotted for rifampicin (RIF) release kinetics from BPU4i-pH8

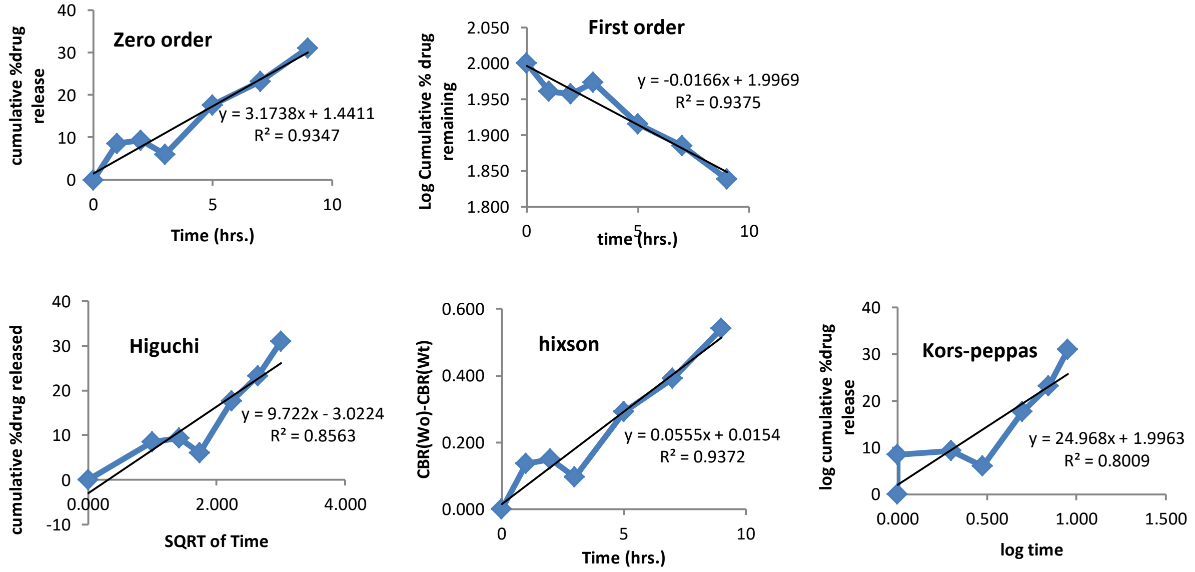

Figure S42 The graphs of Zero order, First order, Higuchi, Hixson-Crowell and Kors-peppas models are plotted for streptomycin (SM) release kinetics from BPU2i-pH2

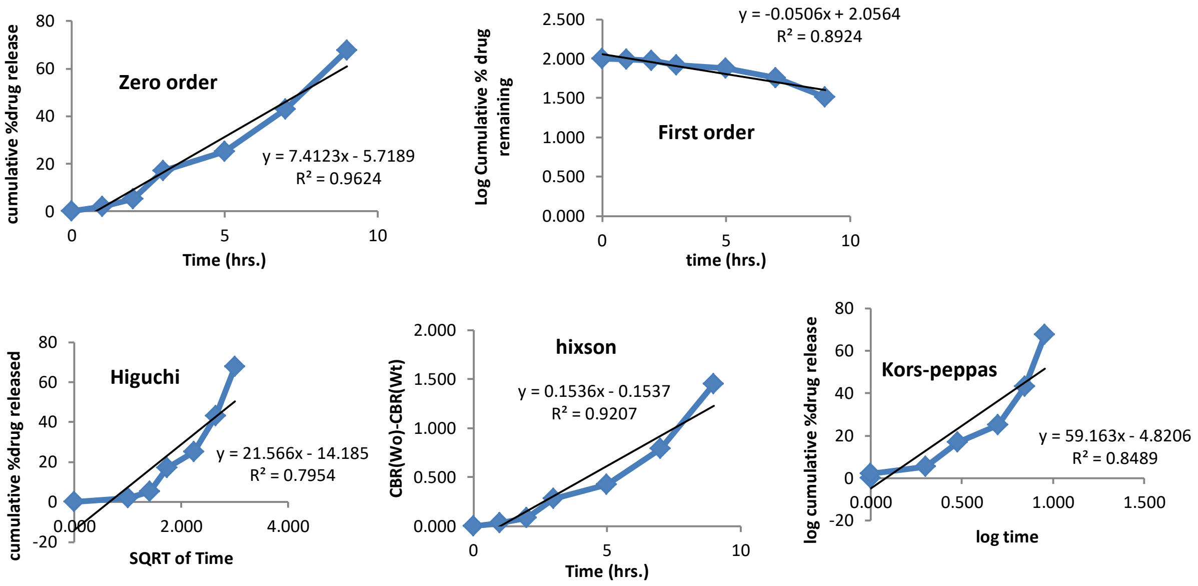

Figure S43 The graphs of Zero order, First order, Higuchi, Hixson-Crowell and Kors-peppas models are plotted for streptomycin (SM) release kinetics from BPU7i-pH8

References for mathematical model graph plotting of Figure No.S29-S43:

1. T. Higuchi, J. Pharm. Sci., 1961, **50**, 874–875. *J. Pharm. Sci*., 1963, **52**, 1145–1149.
2. R. W. Korsmeyer, R. Gurny, E. M. Doelker, P. Buri and N. A. Peppas, *Int. J. Pharm.*, 1983, **15**, 25–35.
3. P. L. Ritger and N. A. Peppas, *J. Controlled Release*, 1987, **5**, 23–36.
4. N. A. Peppas, *Pharm. Acta Helv*., 1985, **60**, 110–111.
5. T. Hayashi, H. Kanbe, M. Okada, M. Suzuki, Y. Ikeda, Y. Onuki, T. Kaneko and T. Sonobe, *Int. J. Pharm.*, 2005, **304**, 91–101.
6. S. Budiasih, K. Jiyauddin, N. Logavinod, M. Kaleemullah, Jawad, A. D. Samer, A. Fadli and Y. Eddy, UK *J. Pharm. Biosci.*, 2014, **2**, 54–61.
7. C. G. M. Heijnen, G. R. M. M. Haenen, F. A. A. Van Acker, W. J. F. Van der Vijgh and A. Bast, *Toxicol. In Vitro*, 2001, **15**.

Table-S2 The loading efficiency and cumulative release percentage (CR%) of nanopolyurethanes (SNPU**2i-7i**) baulkpolyurethanes (SBPU**2i-7i**)

Entry	Structure of ATDs carriers and Code	Loaded - ATDs	LE (%)	CR (%)	
				pH 2	pH 8
1	**nano polyurethane** **NPU2i** 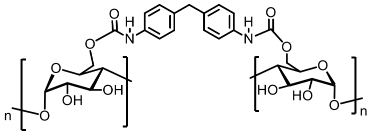	PZA	86.4	37.11	90.2
		INH	60.4	62.7	50.1*
		RIF	80.1	37.9	50.5
		SM	68.0	-	92.7*
2	**nano polyurethane** **NPU3i** 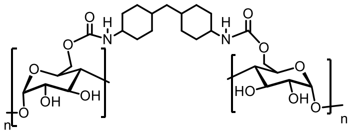	PZA	87.8	33.4	35.2
		INH	62.9	67.3	68.3
		RIF	78.9	16.0	22.6
		SM	93.3	-	75.1
3	**nano polyurethane** **NPU4i** 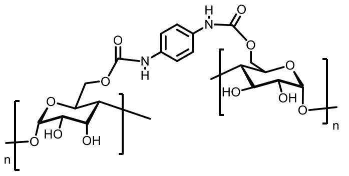	PZA	86.8	21.7	28.6
		INH	63.6	67.3	77.3
		RIF	78.9	-	-
		SM	97.3	-	-
4	**nano polyurethane** **NPU5i** 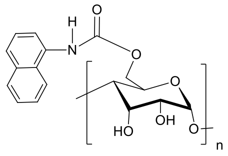	PZA	86.2	92.59	69.3
		INH	65.0	98.8*	89.7*
		RIF	77.9	-	-
		SM	85.3	-	-
5	**nano polyurethane** **NPU6i** 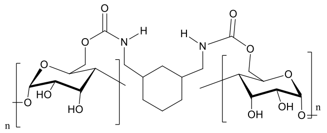	PZA	85.5	30.87	53.5
		INH	65.2	37.3	38.7
		RIF	78.5	10.7	15.7
		SM	85.3	-	-
6	**nano polyurethane** **NPU7i** 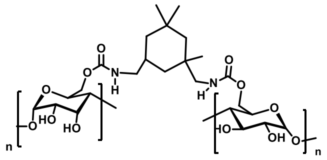	PZA	88.7	36.2	32.4
		INH	61.3	88.3*	94.4*
		RIF	78.5	-	-
		SM	84.6	-	-
7	**bulk polyurethane** **BPU2i** 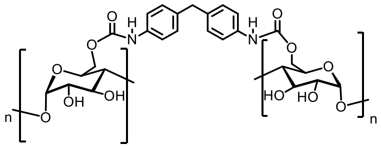	PZA	88.3	32.9	26.5
		INH	68.7	74.6*	85.9*
		RIF	82.5	70.0*	98.7*
		SM	93.9	90.9	75.6*

8	**bulk polyurethane BPU3i** 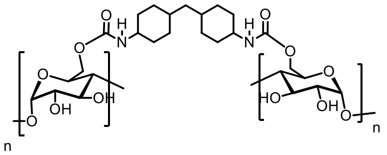	PZA	88.3	22.6	21.3
		INH	66.5	69.0	81.9*
		RIF	76.1	73.8*	92.9*
		SM	90.6	57.1	92.9*
9	**bulk polyurethane BPU4i** 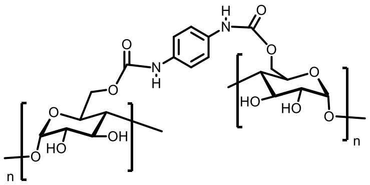	PZA	56.5	11.3	11.7
		INH	59.6	94.2*	96.4*
		RIF	76.1	73.8*	92.9
		SM	84.0	71.5	87.7
10	**bulk polyurethane BPU5i** 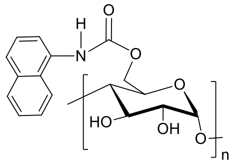	PZA	88.2	45.7	30.0
		INH	57.9	92.2*	89.0*
		RIF	79.4	92.6*	85.0*
		SM	92.3	92.6*	25.2*
11	**bulk polyurethane BPU6i** 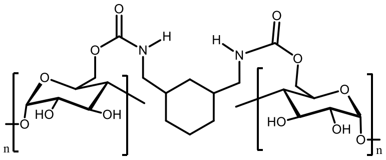	PZA	88.1	3.53	-
		INH	57.9	78.7*	65.5
		RIF	79.4	98.0	89.8*
		SM	96.7	98.0*	70.6
12	**bulk polyurethane BPU7i** 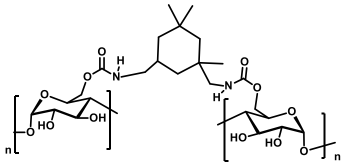	PZA	89.3	37.9	27.6
		INH	65.6	68.1	32.5
		RIF	75.3	97.5	83.2*
		SM	89.7	83.2*	86.1
***burst release profile**					
